# DNA-PK_cs_-dependent phosphorylation of RECQL4 promotes NHEJ by stabilizing the NHEJ machinery at DNA double-strand breaks

**DOI:** 10.1093/nar/gkac375

**Published:** 2022-05-17

**Authors:** Huiming Lu, Junhong Guan, Shih-Ya Wang, Guo-Min Li, Vilhelm A Bohr, Anthony J Davis

**Affiliations:** Department of Radiation Oncology, UT Southwestern Medical Center, Dallas, TX 75390, USA; Department of Radiation Oncology, UT Southwestern Medical Center, Dallas, TX 75390, USA; Department of Radiation Oncology, UT Southwestern Medical Center, Dallas, TX 75390, USA; Department of Radiation Oncology, UT Southwestern Medical Center, Dallas, TX 75390, USA; DNA Repair Section, National Institute on Aging, National Institutes of Health, Baltimore, MD 21224, USA; Department of Radiation Oncology, UT Southwestern Medical Center, Dallas, TX 75390, USA

## Abstract

Non-homologous end joining (NHEJ) is the major pathway that mediates the repair of DNA double-strand breaks (DSBs) generated by ionizing radiation (IR). Previously, the DNA helicase RECQL4 was implicated in promoting NHEJ, but its role in the pathway remains unresolved. In this study, we report that RECQL4 stabilizes the NHEJ machinery at DSBs to promote repair. Specifically, we find that RECQL4 interacts with the NHEJ core factor DNA-PK_cs_ and the interaction is increased following IR. RECQL4 promotes DNA end bridging mediated by DNA-PK_cs_ and Ku70/80 *in vitro* and the accumulation/retention of NHEJ factors at DSBs *in vivo*. Moreover, interaction between DNA-PK_cs_ and the other core NHEJ proteins following IR treatment is attenuated in the absence of RECQL4. These data indicate that RECQL4 promotes the stabilization of the NHEJ factors at DSBs to support formation of the NHEJ long-range synaptic complex. In addition, we observed that the kinase activity of DNA-PK_cs_ is required for accumulation of RECQL4 to DSBs and that DNA-PK_cs_ phosphorylates RECQL4 at six serine/threonine residues. Blocking phosphorylation at these sites reduced the recruitment of RECQL4 to DSBs, attenuated the interaction between RECQL4 and NHEJ factors, destabilized interactions between the NHEJ machinery, and resulted in decreased NHEJ. Collectively, these data illustrate reciprocal regulation between RECQL4 and DNA-PK_cs_ in NHEJ.

## INTRODUCTION

DNA double-strand breaks (DSBs) are cytotoxic DNA lesions that pose an immediate threat to genome stability (1). To cope with DSBs, cells have evolved complex mechanisms collectively termed the DNA damage response (DDR), which includes recognition of the damaged DNA, initiation of cellular signaling cascades, recruitment of DNA repair proteins to the damage site, remodeling of the chromatin near the DSB, activation of cell cycle checkpoints, and repair of the DSB (2,3). The importance of the DDR is unambiguous as defects in the DDR can result in predisposition to cancer, premature aging, and other diseases, including disorders in the nervous, immune, and reproductive systems (1–6). Due to the deleterious nature of DSBs, multiple pathways have evolved to repair this DNA lesion in mammalian cells, including the primary pathways, non-homologous end joining (NHEJ) and homologous recombination (HR), and the minor pathways, alternative end joining (alt-EJ) and single-strand annealing (SSA). NHEJ is a rapid, error-prone DNA repair process that directly re-ligates the two broken DNA strands using a template-independent mechanism and is active in all cell cycle phases. HR directs repair by using a homologous DNA sequence as a template to guide error-free restoration of the DNA molecule (7). HR is primarily active in mid-S to early G2 phase of the cell cycle, because an accessible homologous template via the newly synthesized sister chromatid is readily available in these cell cycle phases (6). Alt-EJ and SSA are intrinsically mutagenic repair processes because both typically use microhomologies or homologous repeats to drive repair, resulting in significant deletions (6). Since multiple processes are available to repair DSBs, a number of control mechanisms, including cell cycle stage, post-translational modifications, chromatin status, and DNA end resection, have evolved to determine how each individual DSB is processed and repaired (6).

NHEJ is the major pathway responsible for the repair of ionizing radiation (IR)-induced DSBs and DSBs intentionally generated for V(D)J and class switch recombination during T- and B-cell lymphocyte maturation (5,8–10). Although NHEJ is often characterized as the repair mechanism that simply rejoins the broken DNA ends regardless of the genetic sequence at the break, it is actually a flexible and dynamic process that can respond to variable types of DSBs (5,11,12). NHEJ initiates when the Ku heterodimer, composed of the Ku70 and Ku80, recognizes and rapidly binds to the DSB in a sequence independent manner (13,14). Once bound to the DSB ends, Ku then performs its primary function as a scaffold to recruit the NHEJ machinery to the DNA damage site. In particular, Ku recruits the DNA-PK_cs_ kinase to the damage site where the DNA-PK complex (DNA-PK_cs_-Ku-DNA) is formed (5). Subsequently, DNA-PK_cs_ is activated and initiates a subset of DDR signaling and chromatin remodeling (5,9,15). If the ends of the DSB are not compatible for ligation, different DNA end processing enzymes are utilized, including those that resect DNA ends, fill in gaps, or remove blocking end groups, to facilitate ligation. The enzymes responsible for processing DNA ends for NHEJ, include Artemis (16,17), Polynucleotide Kinase 3′-Phosphatase (PNKP) (18), Aprataxin (19), Aprataxin and PNK-like factor (APLF) (20), Polymerases μ and λ, Werner (WRN) (21–26) and Ku (27). The terminal step in NHEJ is ligation of the broken DNA ends by DNA Ligase IV (LIG4). LIG4 is stabilized by X-ray repair cross-complementing 4 (XRCC4) and XRCC4, XRCC4-life factor (XLF), and APLF stimulate LIG4-mediated ligation, with XRCC4 and XLF promoting re-adenylation of LIG4 (5,28–31).

RECQL4, which belongs to the family of RecQ helicases, plays multiple roles in DNA metabolism as it contributes to DNA replication, DNA repair, telomere maintenance, preservation of mitochondrial DNA, and mitosis (32–35). RECQL4 possesses the highly conserved 3′ to 5′ DNA helicase domain in the middle of the protein and it unwinds multiple DNA substrates *in vitro*, including Y shaped dsDNA structures, D-loops, and bubble structures, but not duplex DNA or Holliday junctions (36,37). Furthermore, RECQL4 has a strong DNA annealing activity (36). The N- and C-terminal regions of RECQL4 differentiate it from the other RECQ family members. The N-terminus contains multiple functional domains, including both nuclear and mitochondrial targeting sequences and a SLD2-like domain, which is important for promoting DNA replication initiation (35,37,38). Furthermore, the N-terminal region of RECQL4 directs multiple protein-protein interactions and is targeted by multiple post-translational modifications, including phosphorylations and acetylations (38). The RECQL4 C-terminal region contains a Zn^2+^ binding motif and two winged-helix motifs (32,38,39). Mutations in RECQL4 cause three autosomal disorders termed Rothmund-Thomson syndrome (RTS), RAPADILINO, and Baller-Gerold syndrome, with all three clinically associated with premature aging and cancer predisposition (40–42). The majority of the mutations found in *RECQL4* that result in the RTS, RAPADILINO, and Baller-Gerold syndrome are in the helicase domain of RECQL4 (43), supporting the importance of helicase activity of RECQL4 in cellular functions. Elevated expression of RECQL4 is commonly found in multiple cancer types and is typically associated with poor survival (38,44,45). Finally, dysfunction of RECQL4 results in sensitivity to multiple DNA damaging agents, causing increased apoptosis and elevated cellular senescence (46–52).

Emerging evidence shows that RECQL4 is an important player in the repair of DSBs (38). It is rapidly recruited to laser-induced DSBs in both G1 and S/G2 cells and can influence the repair of multiple DSB repair pathways (47,53). RECQL4 plays a crucial role in HR-mediated DSB repair as it promotes 5′ DNA end resection in the S/G2 phases of the cell cycle (49,53). Recently, a study reported that RECQL4 regulates the choice between MMEJ and SSA for a subset of DSBs (54). The role of RECQL4 in NHEJ is less well characterized. An early study found that the *Xenopus laevis* orthologue RecQL4 binds to chromatin at DSB sites near the Ku70 binding site and that depletion of RecQL4 from *Xenopus* egg extracts results in decreased repair of DSBs (55). In human cells, depletion of RECQL4 reduces end-joining activity on DNA substrates with either cohesive or non-cohesive ends *in vitro* and NHEJ efficiency as monitored via a GFP reporter plasmid *in vivo* (48). RECQL4 interacts with the Ku70/Ku80 heterodimer through its N-terminal region and promotes Ku70/Ku80 binding to a blunt-ended dsDNA substrate (48). In this study, we aimed to further elucidate the function of RECQL4 in NHEJ. We found that RECQL4 interacts with the DNA-PK complex rapidly after the generation of DSBs. Furthermore, we identified that there is reciprocal regulation between RECQL4 and DNA-PK_cs_, with each modulating the other's dynamics at DSBs and that this is influenced by DNA-PK_cs_-mediated phosphorylation of RECQL4. Finally, the data illustrate that DNA-PK_cs_-mediated phosphorylation of RECQL4 stabilizes the NHEJ machinery at DSBs to promote NHEJ.

## MATERIALS AND METHODS

### Cell Culture

U2OS and HEK293T cells were cultured in Dulbecco's modified Eagle's medium supplemented with 10% fetal bovine serum (FBS) (Sigma), and 1% penicillin/streptomycin (Gibco). HCT116 DNA-PK_cs_ wild-type, DNA-PK_cs_ with one allele deleted (DNA-PK_cs_^+/–^), DNA-PK_cs_ null (DNA-PK_cs_^–/–^), and DNA-PK_cs_ kinase-dead (DNA-PKcs^KD/–^) cell lines were cultured in Hyclone α-minimum Eagle's medium supplemented with 5% FBS, 5% fetal calf serum and 1% penicillin/streptomycin (15). The cells were grown in an atmosphere of 5% CO_2_ at 37°C. During micro-irradiation assay, the cells were maintained in CO_2_-independent medium (Gibco) with 10% FBS. To inhibit the kinase activity of DNA-PK_cs_ or ATM, cells were incubated for 2 h before the experimental with 10 μM NU7441 (SelleckChem) or 10 μM KU55933 (SelleckChem), respectively.

### Knockout and knockdown

RECQL4-knockout U2OS cells were generated using *RECQL4* gRNA (Supplemental Table 1) following an established protocol (56,57). Knockdown of RECQL4 via siRNA (Supplemental Table 1) was performed as previously described (49) and the control siRNA was purchased from Santa Cruz Biotechnology. Knockdown of MRE11 in RECQL4 knockout U2OS cells expressing GFP-RECQL4, was achieved by using siRNA against MRE11 (Supplemental Table 1) as previously described (49).

### Irradiation.

Cells were irradiated with γ-rays generated by a Mark 1 ^137^Cs irradiator (J.L. Shepherd and Associates) at the doses denoted in the figures.

### Subcellular fractionation

The accumulation of DNA damage response proteins to chromatin following IR-induced DNA damage was examined using the Subcellular Protein Fractionation Kit (Thermo Fisher) as previously described (15). The cells were mock-treated or irradiated with 10 Gy, allowed to recover for 10 min, harvested after trypsinization, and then processed with the Thermo Fisher Subcellular Protein Fractionation Kit according to the manufacturer's instructions. The protein concentration of each sample was measured using a Pierce BCA Protein Assay kit (Thermo Fisher). 30 μg of the cytoplasmic fraction and 15 μg of the soluble nuclear extract or chromatin fraction were resolved via SDS-PAGE, and then transferred to a PVDF membrane for immunoblotting using the protocol outlined below.

### Cell proliferation assay and cell cycle analysis

Cell proliferation rates were measured as previously described (15). Distribution of cell cycle for U2OS parental and RECQL4-knockout cells were measured by staining DNA content with propidium iodide (PI) as previously described with some modifications (49). Briefly, 2 × 10^6^ cells were harvested and fixed with cold 70% Ethanol on ice for 4 h and stored at –20°C overnight. After washing with cold phosphate-buffered saline (PBS) twice, the cells were stained with 500 μl PBS containing 0.1% Triton X-100, 200 μg/ml RNase A, and 20 μg/ml PI at 37°C for 15 min, measured by BD FACSCalibur™ flow cytometer, and analyzed using FlowJo (v.10).

### Immunoblotting and antibodies

Immunoblotting was performed as previously described (15). The following antibodies were used in this study: antibodies from Abcam- anti-DNA-PK_cs_ phospho-S2056 (ab124918), anti-MRE11 (ab214), anti-ATM (ab109027), anti-ATM phospho-S1981 (ab81292); antibodies from Cell Signaling Technology—anti-CHK2 phospho-T68 (2197), anti-LIG4 (14649), anti-Artemis (13381), anti-XLF (2854), anti-phospho S/T-Q motif, anti-mouse IgG (HRP-linked) (7076) and anti-rabbit IgG (HRP-linked) (7074); antibodies from Santa Cruz Biotechnology—anti-XRCC4 (sc-271087), anti-XLF (sc-166488), anti-GFP (sc-8334) and anti-Actin (sc-8432); antibodies from Bethyl Laboratories- anti-KAP1 (A300-274A) and anti-KAP1 phospho-S824 (A300-767A); antibodies from Sigma-Aldrich- anti-tubulin (T5168) and anti-FLAG M2 (F1804); anti-phospho-H2AX (S139) (EMD Millipore, 05-636), and anti-Histone H3 antibody (Biolegend, 819411 or EMD Millipore, 07-690). Anti-RECQL4 was purchased from Genetex (GTX55183). In-house produced antibodies include mouse monoclonal antibodies against DNA-PK_cs_ (Clone #25-4), Ku80, and Ku70 (15).

### Immunoprecipitation (IP) assays

IP assays under native conditions were performed as previously described with slight modifications (53). The cells were mock treated or irradiated with 10 Gy and allowed to recover for 10 min. The cells were washed twice with ice cold PBS, harvested, and then lysed using IP lysis buffer (40 mM Tris-HCl pH 7.4, 125 mM NaCl, 2 mM MgCl_2_, 0.2% NP-40, 0.3% Triton X-100, 1× ThermoFisher Halt protease inhibitor cocktail, 1× Sigma-Aldrich phosphatase inhibitor cocktail 2 and 3, 20 U/ml Novagen Benzonase). The lysates were sonicated on ice and then cleared of cellular debris by centrifuging at 20 000 × g for 30 min. 1.5 mg of total protein was incubated with 2 μg RECQL4 antibody (49), 2 μg DNA-PK_cs_ antibody (25-4) or 2 μg normal rabbit IgG (Invitrogen) in the presence of 30 μl Magnetic Protein A/G beads (Thermo Fisher) overnight at 4°C. The following day the beads were washed five times with IP washing buffer (20 mM Tris-HCl pH 7.4, 125 mM NaCl, 0.1% Triton 100), and then suspended in 1× SDS sample buffer. The immunocomplexes were resolved via SDS-PAGE and immunoblotting analysis was performed as described above and using the antibodies specified in the figure legends.

IP assays under denatured conditions were performed as previously described with slight modifications in order to detect phosphorylation of RECQL4 *in vivo* (53). The plasmid pCMVtag4a-RQ4, which mediates the expression of 3XFLAG-tagged RECQL4, was transiently transfected in HEK293T cells and the cells were mock treated or irradiated with 10 Gy and allowed to incubate for selected time as indicated in the figure legends (typically 20 min). The cells were lysed in lysis buffer (20 mM Tris pH 8.0, 150 mM NaCl, 2% SDS, 1× phosphatase inhibitor cocktail 2, 1× protease inhibitor cocktails), heated at 95°C for 10 min, and sonicated. The lysate was then diluted 10 times with dilution buffer (20 mM Tris pH 8.0, 150 mM, 1% NP-40, 2 mM EDTA, 1× phosphatase inhibitor cocktails 2, 1× protease inhibitor cocktails), and incubated on a rotor at 4 °C for 1 h. After removing cell debris by centrifugation, the lysates were incubated with magnetic M2 FLAG beads (Sigma-Aldrich) overnight at 4°C. Beads were washed three times with Washing buffer 1 (20 mM Tris pH 8.0, 1 M NaCl, 1 mM EDTA, 1% NP-40) and then mixed with 2× SDS sample buffer. The samples were resolved via SDS-PAGE, followed by immunoblotting. Samples were treated with lambda phosphatase (New England Biosciences) at 30°C for 30 min to confirm phosphorylation of RECQL4.

### Laser micro-irradiation and live cell imaging

U2OS cells expressing YFP-tagged Ku80 were constructed in the previous study (58). U2OS cells expressing YFP-tagged DNA-PK_cs_ was generated by selecting single U2OS cells colony with 500 μg/mL G418 after plasmid transfecting using Lonza nucleofector with Solution V and Program X-001. To observe RECQL4′s role in accumulation/retention of DNA-PK_cs_ and Ku80 at laser-induced DSBs, knockdown of RECQL4 was performed as described above and the cells were used for laser micro-irradiation experiments. To assess the accumulation/retention of XRCC4 and XLF to laser-generated DSBs, plasmids expressing GFP-tagged XRCC4 or XLF were transfected into U2OS parental, RECQL4 knockout cells or RECQL4 knockout cells stably expressing 3XFLAG-tagged wild-type RECQL4 or the RECQL4 helicase-dead mutant lysine 508 to methionine (K508M) (mutant named RQ4KM for short)(36) using Lonza Solution V with Program X-001 via the manufacturer's instructions. 24 hr post-transfection the cells were used for the micro-irradiation assays. Recruitment of GFP/YFP-tagged DNA-PK_cs_, RECQL4, XRCC4 and XLF in response to DSB induction were examined following laser micro-irradiation with a Carl Zeiss Axiovert 200M microscope with a Plan-Apochromat 63×/NA 1.40 oil immersion objective (Carl Zeiss) as previously described (15). Briefly, the cells were seeded on a 35 mm glass-bottomed dish (Mattek) and incubated with 10 μM BrdU. 24 h later, the medium was replaced with CO_2_-independent medium and placed in a chamber on the microscope that was set at 37°C. To generate laser-induced DSBs, a 365-nm pulsed nitrogen laser (Spectra-Physics, Catalog #VSL337NDS2, purchased in May 2020) was set at 80% of maximum power output and micro-irradiation was performed using the pulsed nitrogen laser. Time-lapse images were taken using an AxioCam HRm camera (Carl Zeiss). Carl Zeiss Axiovision software (v4.91) was used to measure fluorescence intensities of the micro-irradiated and control areas, and the resulting intensity of irradiated area was normalized to non-irradiated control area to obtain the alteration of the interested proteins as described previously (15,53).

### DNA end bridging assay

To make the GC36 substrate, the biotinylated oligonucleotide Biotin-GC36-top and its complementary oligonucleotide GC36-bottom were annealed at a ratio of 1:1. To make radiolabeled GT50 substrate, oligonucleotide GT50-top and GT50-bottom were annealed and the double-stranded DNA (dsDNA) was labelled with [γ-32P] ATP using T4 polynucleotide kinase (NEB). The oligonucleotide sequences for GC36 and GT50 top and bottom are in Supplementary Table S1. Proteins used in this assay are shown in Supplementary Figure S1 and were prepared in the previously published papers, including DNA-PK_cs_ (59), Ku70/80 (59,60), RECQL4 (49) and RPA (61). DNA end synapsis assay was performed as described previously (62). Briefly, 10 μl streptavidin-coated agarose beads (Thermo Fisher, 20349#) were washed twice with 100 μl Wash buffer (10 mM HEPES pH 7.4, 0.1 mM dithiothreitol (DTT), 1 mM EDTA, 5% glycerol and 1 mg/ml BSA) and then incubated with 1 μM Biotin-GC36 dsDNA in 15 μl binding buffer (Wash buffer supplemented with 150 mM NaCl, 2 mM ATP) for 10 min. Radiolabeled GT50 dsDNA (0.1 μM) and 1 μg DNA-PKcs/Ku (∼120 nM), 0.5 μg RECQL4 (∼200 nM) or 0.5 μg RPA (200 nM) were added and incubated at room temperature for 20 min. The beads were washed three times with 400 μl wash buffer, resuspended with 20 μl buffer and then dotted on nylon membrane. After UVC crosslink for 7 min, the membrane was seated with Fujifilm and visualized by autoradiography. Three independent experiments were performed and quantified by ImageJ.

### *In vitro* phosphorylation and mass spectrometry

To identify DNA-PK-mediated RECQL4 phosphorylation sites, *in vitro* phosphorylation of RECQL4 by DNA-PK was performed by incubating 1 μg recombinant RECQL4 protein (36) with 50 Unit DNA-PK (Promega) in the kinase reaction buffer (50 mM HEPES 7.4, 100 mM KCl, 10 mM MgCl_2_, 1 mM ATP, 0.2 mM EGTA, 0.1 mM EDTA, 1 mM DTT, 10 μg/mL sheared Herring sperm DNA (Sigma) at 30°C for 10 min. The reaction was stopped by incubating the sample at 100°C for 5 min after mixing with SDS sample buffer, and the proteins were separated via SDS-PAGE. After Coomassie blue staining with Simply Blue Safe Stain (Invitrogen), the band containing RECQL4 was sliced and submitted for identification of phosphorylation sites by mass spectrometry analysis by the Taplin Mass Spectrometry Facility at Harvard University.

For analysis of DNA-PK-mediated phosphorylation of RECQL4, pCMVtag4a-RQ4-siR or pCMVtag4a-RQ4-6A-siR were transfected to HEK293T cells, and 3XFLAG-tagged RECQL4 proteins were purified and treated with lambda phosphatase on beads as previously described (53). The beads were divided into two parts, one mixed with DNA-PK in the reaction buffer as described above, and the other one set as control. After reaction, the proteins were resolved via SDS-PAGE, followed by immunoblotting with anti-phospho S/T-Q antibody.

### Cell cycle synchronization

Enrichment of cells at G1 phase and S/G2 were achieved by double thymidine block as previously described (53).

### Site-directed point mutagenesis

Site-directed mutagenesis was performed using PCR to substitute S27, S101, T116, S180, S326 and T336 on RECQL4 with alanine using the pCMVtag4a-RQ4 as a template (53). The primers are listed in Supplementary Table S1. The mutated RECQL4 was designated as RECQL4-6A, which were expressed with 3XFLAG tagged using plasmid pCMVtag4a or GFP-tagged using pEGFP-N1.

### NHEJ and HR reporter assays

NHEJ and HR reporter assays were performed using U2OS EJ5 cells and DR-GFP cells (a gift from Dr. Jeremy Stark at City of Hope), respectively (53,63). The endogenous RECQL4 was depleted using siRNA-mediated knockdown as mentioned above. Three days after siRNA transfection, the cells were transfected with siRNA-resistant plasmids that express vector control (pCMVtag4a), wild-type RECQL4 (pCMVtag4a-RQ4-WT), or phospho-dead RECQL4 (pCMVtag4a-RQ4-6A). One day later, for NHEJ, the cells were transfected with 5 μg plasmids expressing I-SceI endonuclease and DsRed. For HR, the cells were transfected with 5 μg plasmids expressing I-SceI endonuclease as previously described (49,64). Subsequently, the cells were examined using a BD FACSCalibur™ flow cytometer and analyzed using FlowJo (v.10). The data are presented with mean ± SEM from three repeats.

### 53BP foci measurement

IR-induced 53BP1 foci kinetics were monitored in G1 cells as previously described with modifications (60,65). Briefly, the cells were seeded on ‘PTFE’ Printed Slides (Electron Microscopy Sciences) and the following day the cells were mock-treated or irradiated with 2 Gy. At different time points after IR (0.5, 1, 3 or 7 hours), the cells were washed twice with ice cold 1× PBS and fixed with 4% paraformaldehyde (in 1× PBS) for 20 min at RT, washed 5 times with 1× PBS, and incubated in 0.5% Triton X-100 on ice for 10 min. Cells were washed 5 times with 1× PBS and incubated in blocking solution (5% goat serum (Jackson Immuno Research) in 1× PBS) for 1h. The blocking solution was replaced with the 53BP1 (ab175933, Abcam) and Cyclin A2 (ab16726, Abcam) primary antibodies (1:1000 dilution for both antibodies) diluted in 5% normal goat serum in 1× PBS and the cells were incubated at 4°C overnight. The next day the cells were washed 5 times with wash buffer (1% BSA in 1× PBS). Next, the cells were incubated with anti-rabbit IgG conjugated with Alexa Fluor 488 (Molecular Probes) and anti-mouse IgG conjugated with Texas Red (Molecular Probes) (1:1000 dilution for both antibodies) secondary antibodies in 1% BSA, 2.5% goat serum in 1× PBS for 1 h in the dark, followed by five washes. After the last wash, the cells were mounted in VectaShield Antifade mounting medium containing 4′,6-diamidino-2-phenylindole (DAPI). Images were acquired using a Zeiss AxioImager fluorescence microscope utilizing a 63× oil objective lens. The 53BP1 foci were only counted in the cells with no Cyclin A staining.

### Clonogenic survival assay

Cell survival curves were obtained by measuring the colony-forming abilities of irradiated cell population as previously described with modifications (53,66). The endogenous RECQL4 was depleted in U2OS cells by siRNA-mediated knockdown as mentioned above. Two days after siRNA transfection, the cells were transfected with siRNA-resistant plasmids that express vector control (pCMVtag4a), wild-type RECQL4 (pCMVtag4a-RQ4-WT), or phospho-dead RECQL4 (pCMVtag4a-RQ4-6A). Two days later, the cells were irradiated at doses of 1, 2, 4 or 6 Gy and then plated on 60-mm plastic Petri dishes. After 10 days, cells were fixed and stained with 0.1% crystal violet in a 100% ethanol solution. Colonies were scored and the mean value for triplicate culture dishes was determined. Cell survival was normalized to plating efficiency of untreated controls for each cell type. The results are presented as mean ± SEM from three experiments.

## RESULTS

### DNA damage induces the interaction between RECQL4 and the DNA-PK complex

**Figure 1.**
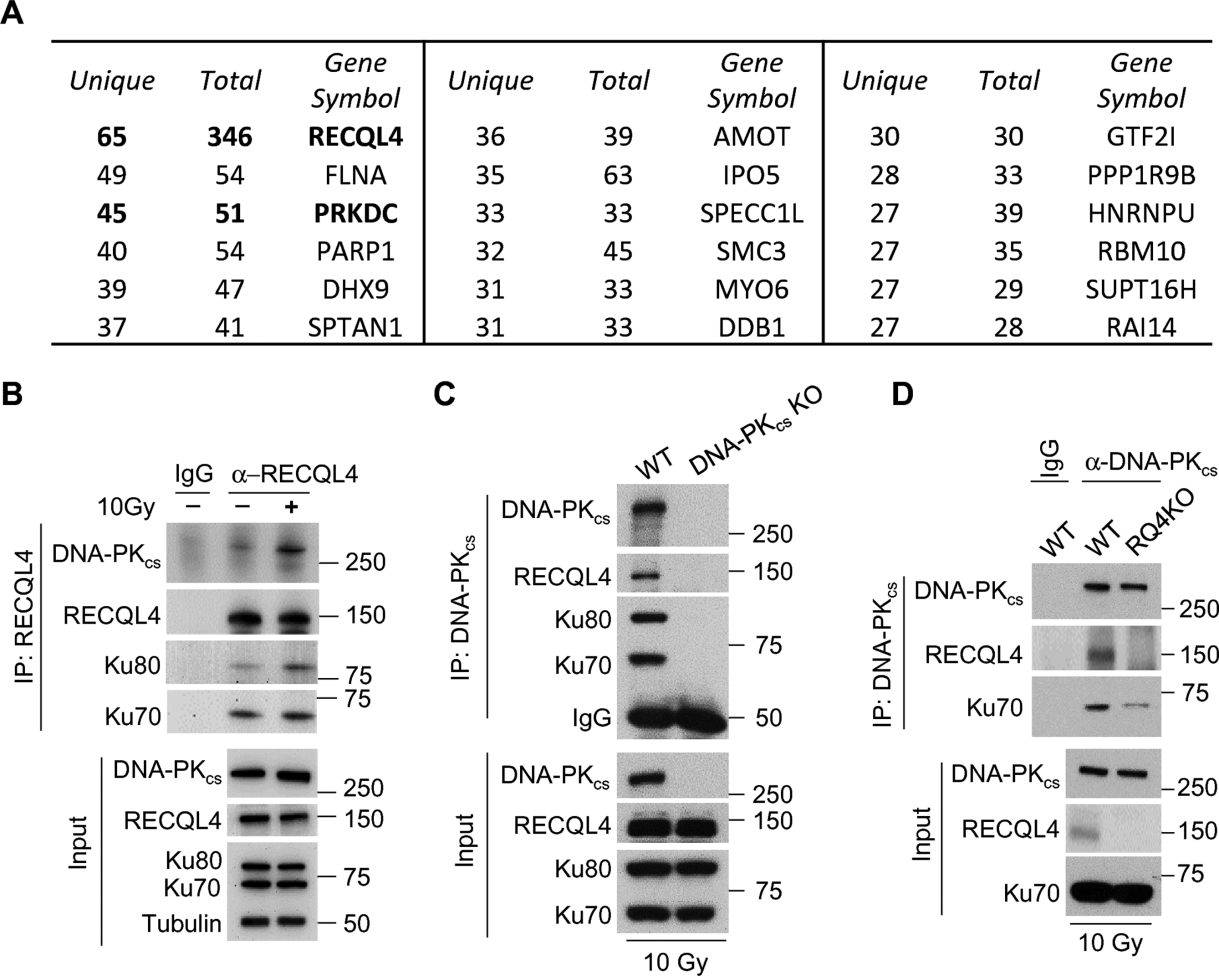
Ionizing radiation stimulates the interaction between RECQL4 and DNA-PK_cs_. (**A**) Identification of RECQL4-associated proteins following DNA damage. U2OS cells were irradiated with 10 Gy of IR, allowed to recover for 10 min, and RECQL4 was immunoprecipiated from whole cell lysates. The samples were resolved via SDS-PAGE, stained, and then analyzed using mass spectrometry analysis. Shown are the top 17 RECQL4-interacting proteins identified in the screen. DNA-PK_cs_ (gene: *PRKDC*) was a top hit in the RECQL4 protein-protein interaction screen. (**B**) DNA damage promotes the interaction between RECQL4 and the DNA-PK complex (Ku70/80 heterodimer and DNA-PK_cs_). U2OS cells were mock treated or irradiated with 10 Gy and allowed to recover for 10 min. Endogenous RECQL4 was immunoprecipitated and its interaction with the DNA-PK complex was assessed via immunoblotting using DNA-PK_cs_, Ku70 and Ku80 antibodies. (**C**) DNA-PK_cs_ was immunoprecipitated from HCT116 wild-type (WT) or DNA-PK_cs_ knockout (DNA-PKcs KO) cells that were treated with 10 Gy of IR and allowed to recover for 10 min. The samples were resolved via SDS-PAGE and the interaction between DNA-PK_cs_ and RECQL4 and Ku70/80 was determined via immunoblotting. (**D**) DNA-PK_cs_ was immunoprecipitated from U2OS wild-type (WT) or RECQL4 knockout (RQ4KO) cells that were irradiated with 10 Gy of IR and allowed to recover for 10 min. The samples were resolved via SDS-PAGE and interaction between RECQL4 and DNA-PK_cs_ and Ku70/80 was determined via immunoblotting.

Previous reports have shown that RECQL4 quickly localizes to laser-generated DSBs (<10 s post-microirradiation), indicating that it plays an initial role in the cellular response to DSBs (47,49,53). In this study, we aimed to mechanistically elucidate the function of RECQL4 in the early response to DSBs. To investigate this, we performed an immunoprecipitation-mass spectrometry experiment to identify RECQL4 interacting proteins at an early time point (10 min) post-irradiation (Supplementary Table S2). One of the top hits in the screen was the NHEJ factor, DNA-PK_cs_ (gene: *PRKDC*) (Figure 1A and Supplementary Table S2). Since RECQL4 interacts with the DNA-PK complex factors Ku70 and Ku80, it suggests that RECQL4 interacts with the DNA-PK complex (48,53). To confirm this, we performed co-immunoprecipitation assays using RECQL4 or DNA-PK_cs_ antibodies. DNA-PK_cs_, Ku70, and Ku80 co-immunoprecipitated with RECQL4 and the interaction was induced by DNA damage (Figure 1B). RECQL4 co-immunoprecipitated with DNA-PK_cs_ and this interaction is specific as the RECQL4-DNA-PK_cs_ interaction was abrogated in DNA-PK_cs_ and RECQL4 knockout cells and the interaction is not dependent on DNA as the immunoprecipitation samples were treated with benzonase to remove DNA to block nonspecific DNA-protein interactions (Figure 1C and D). Collectively, the data show RECQL4 interacts with the DNA-PK complex rapidly after the generation of DSBs.

### RECQL4 promotes DNA end bridging by the DNA-PK complex *in vitro*

**Figure 2.**
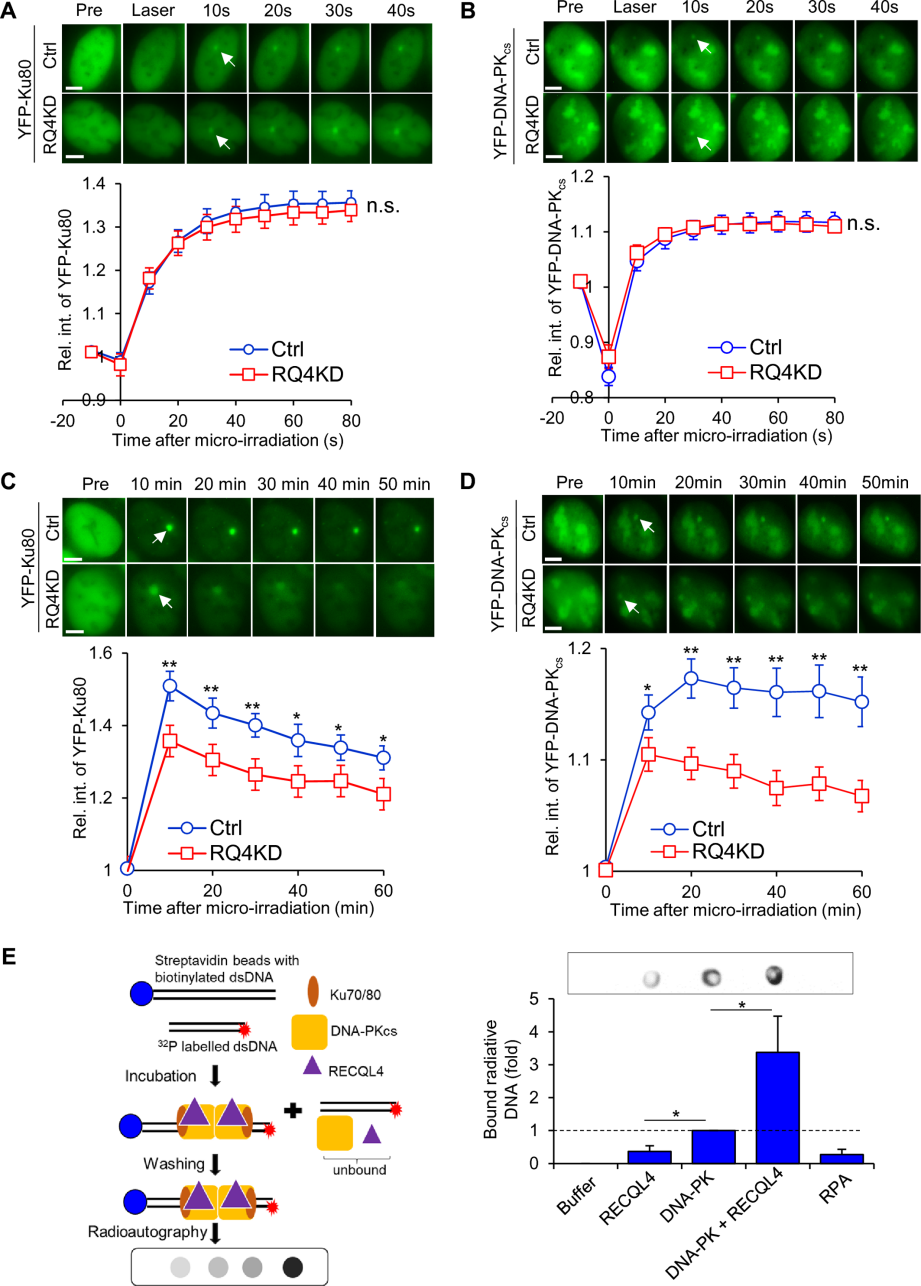
RECQL4 promotes DNA-PK-mediated DNA end bridging. (**A, B**) Initial recruitment of YFP-tagged Ku80 (**A**) and DNA-PK_cs_ (**B**) to laser-induced DSB is not affected by knockdown of RECQL4 in U2OS cells. Relative fluorescent intensity of YFP-tagged Ku80 and DNA-PK_cs_ in U2OS cells treated with *RECQL4* siRNAs (RQ4KD) or control siRNAs (Ctrl) following micro-irradiation are presented as mean ± standard error of the mean (SEM). The knockdown efficiency is shown in Supplementary Figure S2B and D. Samples analyzed were 11 Ctrl and 13 RQ4KD cells for YFP-tagged Ku80 and 13 Ctrl and 19 RQ4KD cells for YFP-tagged DNA-PK_cs_. Student's t-test (two-sided) was performed to assess statistical significance (n.s. = not significant). (**C, D**) Depletion of RECQL4 attenuates the accumulation of Ku80 (**C**) and DNA-PKcs (**D**) at laser-induced DSBs. Relative fluorescent intensity of GFP-tagged Ku80 and DNA-PK_cs_ following micro-irradiation are presented as mean ± SEM. The knockdown efficiency is shown in Supplementary Figure S2B and D. Samples analyzed were 19 Ctrl siRNA treated cells and 17 RQ4KD cells for YFP-tagged Ku80, and 12 Ctrl and 18 RQ4KD cells for YFP-tagged DNA-PK_cs_. Student's t-test (two-sided) was performed to assess statistical significance (* *P*< 0.05 and ** *P*< 0.01). (**E**) RECQL4 stimulates end bridging of dsDNA end by DNA-PK *in vitro*. The indicated purified proteins were incubated with a biotin-labeled 36 bp dsDNA with one end linked to streptavidin beads and a 50 bp dsDNA with radioactive ^32^P labeled, the DNA-protein complex were pulled down, washed, and dotted on a nylon membrane for radiography. The signal indicates amount of free dsDNA (50bp) bridged with beads-bound dsDNA (36bp) by proteins. The data were generated from three independent experiments and presented as mean ± SEM. Two-sided student's t-test (two-sided) was performed to assess statistical significance (* *P*< 0.05).

As RECQL4 interacts with the DNA-PK complex immediately after DSB induction, we next examined if RECQL4 regulates DNA-PK at DSBs. First, we assessed if RECQL4 modulates the dynamics of the DNA-PK complex following DNA damage. Knockdown of RECQL4 does not affect the initial recruitment of the Ku heterodimer (Figure 2A and Supplementary Figure S2A and B) or DNA-PK_cs_ (Figure 2B and Supplementary Figure S2C and D) to laser-induced DSBs. DNA-PK_cs_ is activated, as monitored by autophosphorylation at serine 2056, in response to IR-induced DNA damage at early time points in RECQL4 knockout and knockdown cells (Supplementary Figure S3A and B). Moreover, initiation of the DNA damage response (DDR) signalling pathways is unaffected, as the DNA-PK_cs_ and ATM substrates, H2AX, KAP1 and CHK2 are phosphorylated similarly in control and RECQL4 knockdown and knockout cells (Supplementary Figure S3A and B). These data illustrate that RECQL4 does not affect the initial recruitment of DNA-PK to DSBs, and other key proteins involved in the initiation of the DDR. However, Ku and DNA-PK_cs_ accumulation/retention at DSBs was significantly decreased in RECQL4 knockdown cells compared to control cells, suggesting that RECQL4 is required for the stabilization of the DNA-PK complex at DSBs (Figure 2C and D and Supplementary Figure S2B and D). To further investigate the role of RECQL4 in regulating the DNA-PK complex at DSB ends, we examined if RECQL4 regulates DNA-PK-dependent DNA end bridging. In support of previous reports, we observed that DNA-PK promotes DNA end synapsis *in vitro* (62,67–72) (Figure 2E). RECQL4 alone can only slightly direct DNA end bridging, but it strongly stimulated DNA end synapsis by the DNA-PK complex (Figure 2E). These results illustrate that RECQL4 regulates the dynamics of the DNA-PK complex at DNA damage sites and ameliorates DNA-PK-dependent DNA end bridging.

### RECQL4 promotes the stabilization of the NHEJ machinery at DSBs

**Figure 3.**
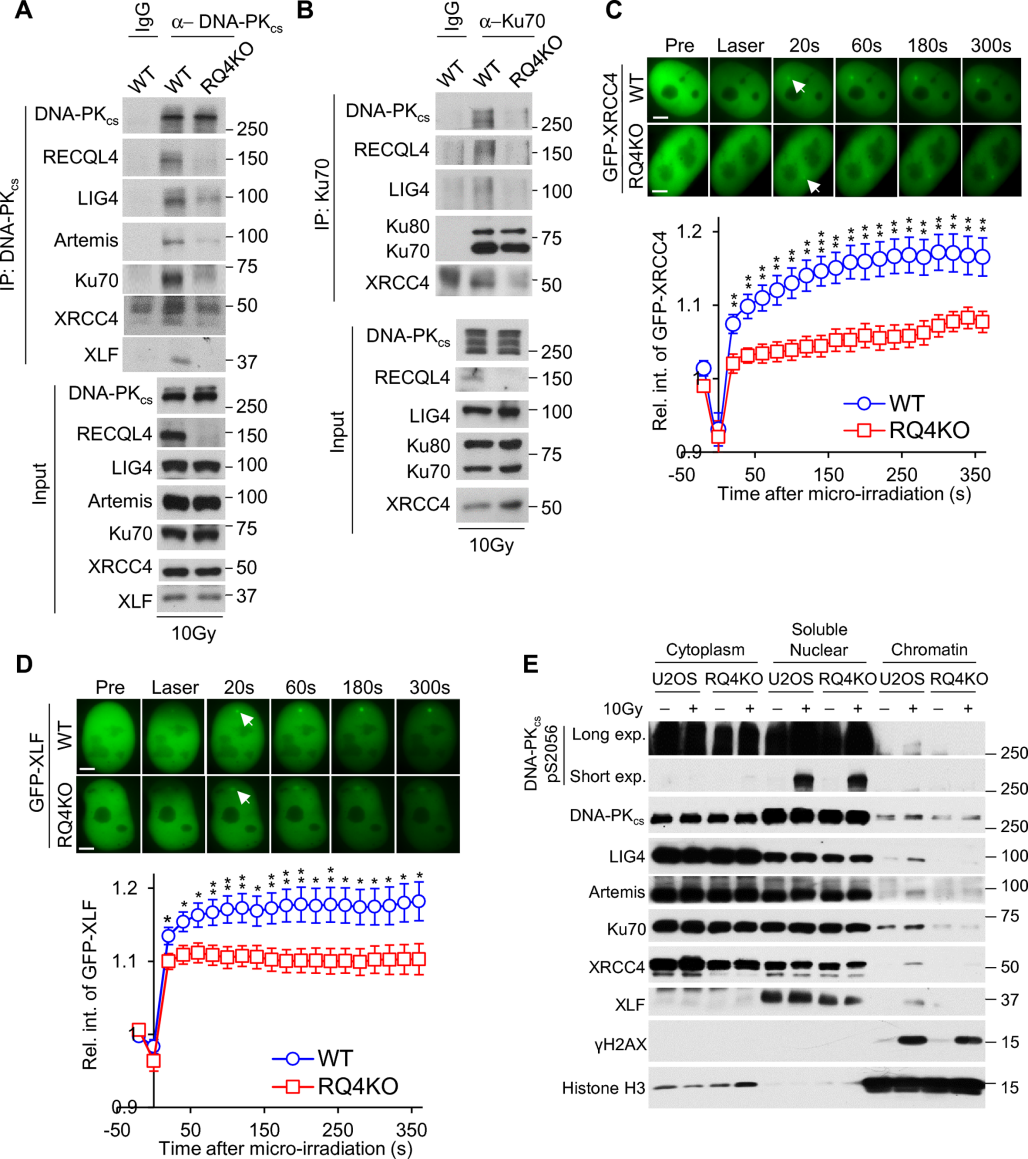
RECQL4 promotes stabilization of NHEJ machinery at DSBs. (**A, B**) NHEJ protein–protein interactions are significantly decreased in the absence of RECQL4. DNA-PK_cs_ (**A**) and Ku70 (**B**) were immunoprecipitated from U2OS wild-type (WT) or RECQL4 knockout (RQ4KO) cells that were treated with 10 Gy of IR and allowed to recover for 10 minutes. The samples were resolved via SDS-PAGE and interactions between DNA-PK_cs_ and Ku70 with NHEJ factors (LIG4, XRCC4, XLF and/or Artemis) and RECQL4 were determined via immunoblotting. (**C**, **D**) RECQL4 promotes recruitment of XRCC4 and XLF to laser-generated DSBs. Relative fluorescent intensity of GFP-tagged XRCC4 (**C**) and XLF (**D**) in WT or RQ4KO cells following micro-irradiation are presented as mean ± standard error of the mean (SEM). For XRCC4, the results were calculated from 7 WT and 12 KO cells and 9 WT and 10 RQ4KO cells for XLF. Student's *t*-test (two-sided) was performed to assess statistical significance (* *P*< 0.05, ** *P*< 0.01 and *** *P*< 0.001). (**E**) Recruitment of NHEJ core factors to chromatin after IR is attenuated in RECQL4 knockout (RQ4KO) cells. U2OS wild-type (WT) and RQ4KO cells were mock-treated or irradiated with a dose of 10 Gy and allowed to recover for 10 min. Subsequently, cytoplasmic, soluble nuclear, and chromatin fractions were isolated for immunoblotting to examine the recruitment of proteins listed in the figure to the chromatin after irradiation.

RECQL4 has been implicated to participate in NHEJ, but its functions in this DSB repair pathway is unclear (48,55). Our results show that RECQL4 promotes the stabilization of the DNA-PK complex at DSBs and DNA-PK-dependent DNA end synapsis, which suggest that RECQL4 is required for the stabilization of the NHEJ machinery at DSBs. To examine this, we immunoprecipitated DNA-PK_cs_ or Ku70 from irradiated control or RECQL4 knockout cell lysates and assessed co-immunoprecipitation of multiple NHEJ factors. The pairwise interactions between DNA-PK_cs_ and Ku70 with each other, and with DNA ligase IV (LIG4), XRCC4, XLF, and Artemis were all attenuated in RECQL4 knockout cells compared to control cells (Figure 3A and B). This decrease is not due to altered expression of the NHEJ factors, cell cycle distribution, or cell proliferation rate, as similar protein amounts were observed in control and RECQL4 knockout cells (Input lanes of Figure 3A and B and Supplementary Figure S3B), and cell cycle distribution and cell proliferation remain unchanged after RECQL4 knockout (Supplementary Figure S3C and D). Next, we investigated if the accumulation of NHEJ factors to laser-generated DSBs is affected in RECQL4 knockout cells. We observed a significant decrease in the recruitment and accumulation of GFP-tagged XRCC4 and XLF to DSBs in RECQL4 knockout cells compared to control cells (Figure 3C and D). These results are supported by the experiments showing that accumulation of the NHEJ factors, DNA-PK_cs_, Ku70, LIG4, XRCC4 and XLF to the chromatin fraction following DNA damage is markedly decreased in RECQL4 knockout cells compared to control cells (Figure 3E). Similar results were observed in RECQL4 knockdown cells (Supplementary Figure S4). As RECQL4 possesses 3′ to 5′ DNA helicase activity, we next examined if the DNA unwinding of RECQL4 is required for the ability of the protein to promote the stabilization of the NHEJ machinery at DSBs. To assess this, we complemented RECQL4 knockout cells with 3XFLAG-tagged wild-type RECQL4 or the helicase-dead (K508M) RECQL4 (RQ4KM) (36) and then examined pairwise interactions between the NHEJ factors following immunoprecipitation of DNA-PK_cs_ and by monitoring the recruitment of GFP-tagged XRCC4 to laser-induced DSBs. As shown in Supplementary Figures S5A and B, IR-induced pairwise interactions between DNA-PK_cs_ and Ku80, XRCC4 and XLF and recruitment of GFP-XRCC4 to laser-generated DSBs are similar in RECQL4 wild-type and helicase-dead mutant cells. These results implicate that the helicase activity is not important for RECQL4′s ability to facilitate stabilization of NHEJ machinery at DSBs. Together, the data illustrate RECQL4 promotes the stabilization of the NHEJ machinery at DSBs and that this is not dependent on the helicase activity of RECQL4.

### DNA-PK_cs_ kinase activity promotes the accumulation of RECQL4 at DSBs

**Figure 4.**
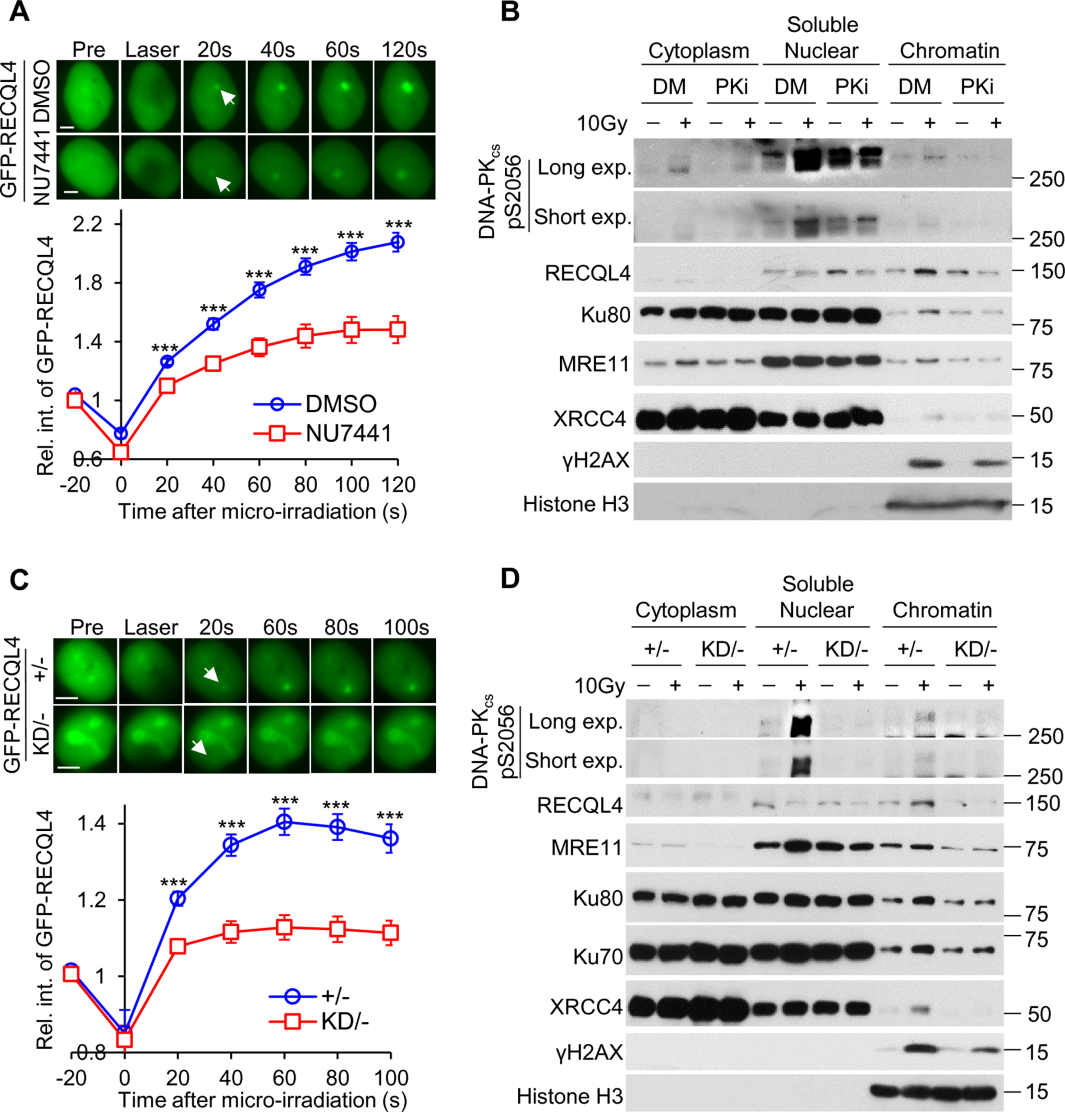
The kinase activity of DNA-PK_cs_ promotes the accumulation of RECQL4 at DSBs. (**A**) Inhibition of DNA-PK_cs_ attenuates accumulation of RECQL4 at laser-induced DSBs. U2OS RECQL4 Knockout cells stably expressing GFP-tagged RECQL4 were pretreated either with DMSO or 10 μM NU7441 for 2 h, and subsequently laser micro-irradiation assays were performed. Relative fluorescent intensity of GFP-tagged RECQL4 following micro-irradiation are presented as mean ± standard error of the mean (SEM). Samples analyzed were 15 for DMSO-treated and 16 NU7441-treated cells. Student's *t*-test (two-sided) was performed to assess statistical significance (*** *P*< 0.001). (**B**) Inhibition of DNA-PK_cs_ reduces the recruitment of RECQL4 to the chromatin fraction following IR. U2OS cells were pretreated with 10 μM NU7441 or DMSO for 2 h, mock-treated or irradiated with a dose of 10 Gy, and allowed to recover for 10 min. Subsequently, cytoplasmic, soluble nuclear and chromatin fractions were isolated for immunoblotting to examine the recruitment of proteins listed in the figure to the chromatin after irradiation. (**C**) Accumulation of GFP-tagged RECQL4 in HCT116 DNA-PK_cs_ kinase-dead (KD/–) cells is reduced compared to that in control DNA-PK_cs_^+/–^ cells (+/–). Relative fluorescent intensity of GFP-tagged RECQL4 following micro-irradiation are presented as mean ± standard error of the mean (SEM). Samples analyzed were 8 for +/– and 8 for KD/– cells. Student's t-test (two-sided) was performed to assess statistical significance (*** *P*< 0.001). **(D)** Recruitment of RECQL4 to the chromatin fraction is decreased in KD/– cells compared to +/– cells. KD/– and +/– cells were mock-treated or irradiated with a dose of 10 Gy and allowed to recover for 10 minutes. Subsequently, cytoplasmic, soluble nuclear and chromatin fractions were isolated for immunoblotting to examine the recruitment of proteins listed in the figure to the chromatin after irradiation.

Next, we examined if DNA-PK_cs_ kinase activity regulates RECQL4 in the early response to DNA damage. First, we determined if the recruitment of RECQL4 to DSBs is modulated by DNA-PK_cs_ kinase activity. We found that inhibition of DNA-PK_cs_ using the selective inhibitor NU7441 significantly attenuated the recruitment of GFP-tagged RECQL4 to laser-generated DSBs (Figure 4A and Supplementary Figure S6A). Consistent with this result, RECQL4 accumulation to the chromatin fraction was decreased in NU7441-treated cells compared to control cells following irradiation (Figure 4B). Furthermore, we examined the recruitment of RECQL4 to DNA damage in DNA-PK_cs_ wild-type (DNA-PKcs^+/–^) and kinase dead (DNA-PKcs^KD/–^) cell lines. Similar to the results using the DNA-PK_cs_ inhibitor, the recruitment of GFP-tagged RECQL4 to laser-induced DSBs and recruitment to the chromatin fraction following IR was significantly decreased in DNA-PKcs^KD/–^ cells compared to DNA-PKcs^+/–^ cells (Figure 4C and D). The attenuation of RECQL4 is specific for DNA-PK_cs_ activity as pretreatment with the ATM inhibitor KU55933 did not alter the recruitment of GFP-RECQL4 to laser-induced DSBs (Supplementary Figure S6B). This result is consistent with previous findings that the recruitment of RECQL4 to DSBs was not affected by inhibition of ATM (53) or in ATM-deficient fibroblasts (47). We previously found that knockdown of MRE11 reduced accumulation of RECQL4 at laser-induced DSBs (49). To determine if DNA-PK_cs_-dependent recruitment of RECQL4 to DSBs is influenced by MRE11 or vice versa, we monitored the recruitment of GFP-tagged RECQL4 in control cells or those with MRE11 siRNAs, NU7441, or the combination of MRE11 siRNAs with NU7441. As shown in Supplementary Figure S6C, knockdown of MRE11 significantly reduces the accumulation of GFP-tagged RECQL4 to laser-induced DSBs compared to control cells, which is consistent with previously published results (53). Furthermore, treatment with NU7441 results in a significant decrease in accumulation of GFP-tagged RECQL4 to laser-generated DSBs compared to the control cells and the MRE11 knockdown cells. Interestingly, we found accumulation of RECQL4 to DSBs is similar in the NU7441 treated cells and the cells co-treated with MRE11 siRNA + NU7441. Together, the data show that the kinase activity of DNA-PK_cs_, but not ATM, promotes recruitment of RECQL4 to DSBs.

### DNA-PK_cs_ phosphorylates RECQL4

**Figure 5.**
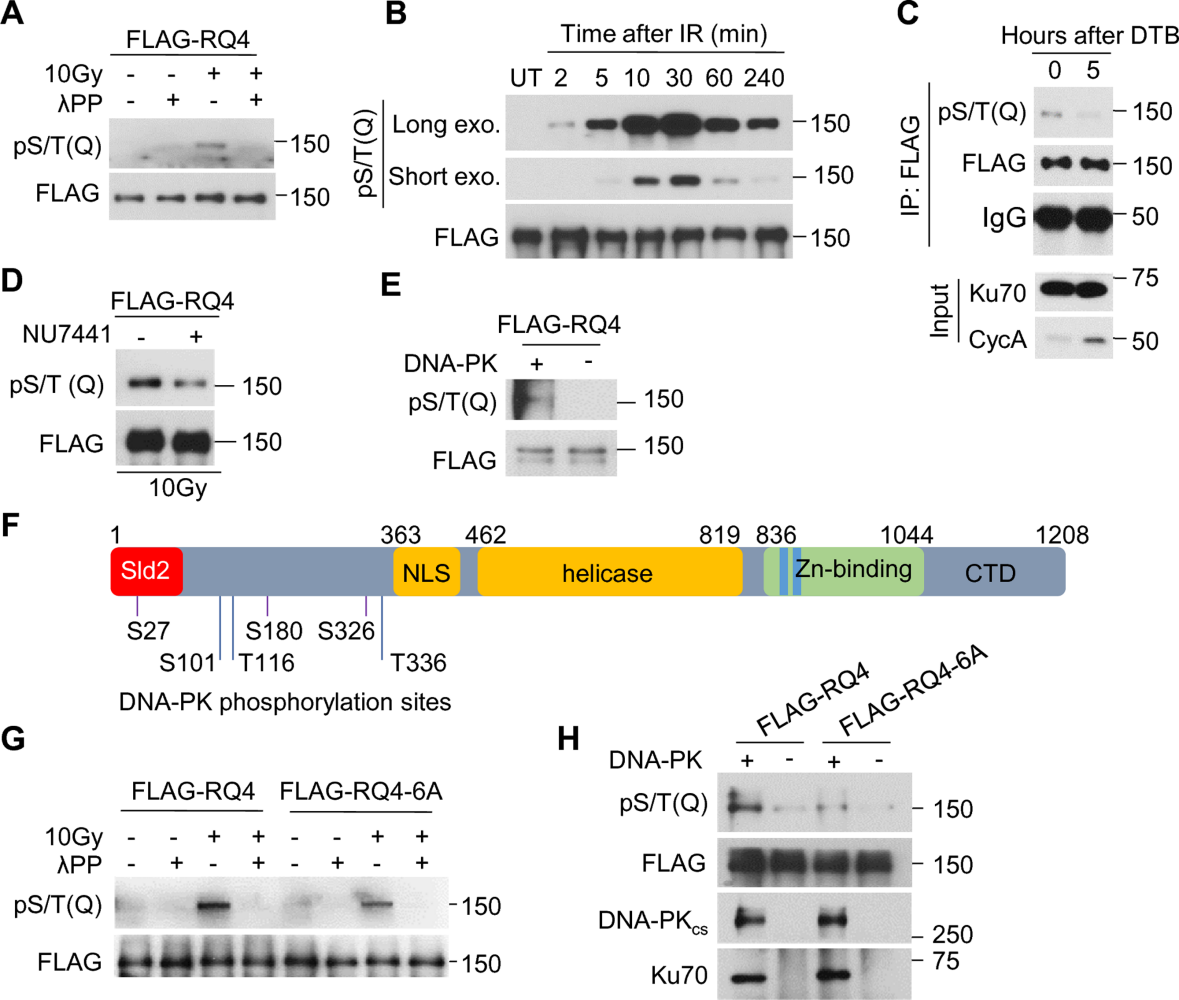
RECQL4 is phosphorylated by DNA-PK. (**A**) RECQL4 is phosphorylated at the S/T-Q motif after treatment with ionizing radiation (IR). HEK293T cells transiently expressing 3XFLAG-tagged RECQL4 were mock treated or irradiated with 10 Gy and allowed to recover for 20 min. The cells were lysed under denaturing conditions and 3XFLAG-tagged RECQL4 was purified using FLAG-M2 beads and subsequently mock treated or treated with lambda phosphatase (λPP). The samples were resolved via SDS-PAGE and immunoblotting was performed using a phospho-S/T-Q motif antibody. (**B**) IR-induced phosphorylation of RECQL4 at the S/T-Q motif is time dependent. HEK293T cells expressing 3XFLAG-tagged RECQL4 were irradiated with 10Gy and lysed at the time points indicated in the figure. Samples were processed and analyzed as stated in (A). (**C**) IR-induced phosphorylation of RECQL4 at the S/T-Q motif is increased in G1 phase of the cell cycle. RECQL4 knockout U2OS cells expressing 3XFLAG-RECQL4 were synchronized to G1/S border by double thymidine block (DTB), and then released in regular medium for 5 hours to enrich S/G2 cells. Phosphorylation at the S/T-Q motif was assessed using the protocol described in (A). (**D**) Phosphorylation of RECQL4 at the S/T-Q motif is attenuated when DNA-PK_cs_ is inhibited. HEK293T cells expressing 3XFLAG-tagged RECQL4 were pretreated with 10 μM NU7441 or DMSO for 2 h, mock-treated or irradiated for 10 Gy, and allowed to recover for 20 min. Samples were processed and analyzed as stated in (A). (**E**) DNA-PK phosphorylates RECQL4 *in vitro*. Purified 3XFLAG-tagged RECQL4 was treated with λPP, and incubated with DNA-PK_cs_, Ku70/80 and DNA in the presence of ATP. Phosphorylation at the S/T-Q motif was assessed with Western blotting. (**F**) DNA-PK_cs_-mediated phosphorylation sites on RECQL4. RECQL4 was phosphorylated by DNA-PK *in vitro* and six phosphorylation sites (S27, S101, T116, S180, S326 and T336) in the N-terminal region of RECQL4 were identified by mass spectrometry analysis. Sld2, Sld2-like domain; NLS, nuclear location signal; Helicase, RecQ helicase domain; Zn-binding, Zn^2+^ binding motif; CTD, C-terminal domain. (**G**) Ablating the DNA-PK_cs_-mediated phosphorylation sites on RECQL4 results in a decrease in IR-induced phosphorylation of RECQL4 at the S/T-Q motif. HEK293T cells transiently expressing 3XFLAG-tagged wild-type RECQL4 (FLAG-RQ4) or phosphorylation-null mutant (FLAG-RQ4-6A) were mock treated or irradiated with 10 Gy and allowed to recover for 20 min. Samples were processed and analyzed as stated in (A). (**H**) DNA-PK_cs_-dependent phosphorylation of RECQL4 is significantly decreased when the six phosphorylation sites are ablated. Purified FLAG-RQ4 and FLAG-RQ4-6A were treated with λPP, eluted from the FLAG-M2 beads, and incubated with DNA-PK_cs_, Ku70/80 heterodimer and DNA in presence of ATP. Phosphorylation at the S/T-Q motif was assessed with Western blotting as described above.

As the kinase activity of DNA-PK_cs_ promotes the recruitment of RECQL4 to DSBs, we postulated that DNA-PK_cs_ phosphorylates RECQL4, and this stabilizes RECQL4 at DSBs. To examine this, we first determined if RECQL4 is phosphorylated in response to IR. Initially, we used the commercially available S/T-Q motif phosphorylation antibody, as DNA-PK_cs_ can phosphorylate substrates at this motif (73,74). RECQL4 was phosphorylated at the S/T-Q motif in response to DNA damage and this signal was lost when the sample was treated with lambda phosphatase (Figure 5A). A time course found that RECQL4 phosphorylation at the S/T-Q motif appears as early as 2 min post-irradiation and peak phosphorylation was reached at 30 min (Figure 5B). The IR-induced phosphorylation of RECQL4 at the S/T-Q motif sites is specific for G1 phase of the cell cycle, as the signal is significantly lost in cells in S phase (Figure 5C). Pretreatment with the DNA-PK_cs_ inhibitor NU7441 resulted in a decrease in RECQL4 phosphorylation at the S/T-Q motif (Figure 5D), indicating that DNA-PK_cs_ phosphorylates RECQL4 at this motif *in vivo*. Moreover, purified DNA-PK phosphorylates RECQL4 *in vitro* (Figure 5E), providing further evidence that RECQL4 is a substrate of DNA-PK_cs_. To identify DNA-PK_cs_-mediated phosphorylation sites, RECQL4 was phosphorylated using purified DNA-PK *in vitro* and the samples were submitted for mass spectrometry analysis. Eight sites were identified (Supplementary Table S3) and six were selected for further study, including three sites with S/T-Q motifs (S27, S180 and S326) and three non-S/T-Q motifs (S101, T116 and T336) (Figure 5F). Interestingly, each of the phosphorylation sites are located in the N-terminal domain of RECQL4, which is the region of the protein that promotes many of RECQL4′s protein-protein interactions (75). To assess if these sites are responsible for the phosphorylation signal observed with the phospho-S/T-Q motif antibody, we mutated the six serine/threonine residues to alanine to ablate phosphorylation (RQ4-6A). Mutating the six sites reduced the IR-induced phosphorylation signal by the phospho S/T-Q motif antibody (Figure 5G), indicating that phosphorylation of RECQL4 at these S/T-Q site(s) occur *in vivo*. However, the phospho-S/T-Q motif signal is not completely lost, suggesting that there are other S/T-Q sites phosphorylated outside of the three identified in our screen or that the antibody recognizes S/T-Q sites phosphorylated independently of DNA-PK_cs_ (Figure 5D and G). To determine if the sites identified in our screen are targeted directly by DNA-PK_cs_, we conducted an *in vitro* phosphorylation assay using DNA-PK and either wild-type RECQL4 or RQ4-6A. DNA-PK_cs_ phosphorylates wild-type RECQL4 but not RECQL4 in which these phosphorylation sites were ablated (Figure 5H), confirming that RECQL4 is targeted by DNA-PK_cs_ at these sites *in vitro*. Collectively, these data demonstrate that RECQL4 is phosphorylated by DNA-PK_cs_ at six sites in the N-terminus of the protein.

### DNA-PK-mediated phosphorylation of RECQL4 promotes NHEJ

**Figure 6.**
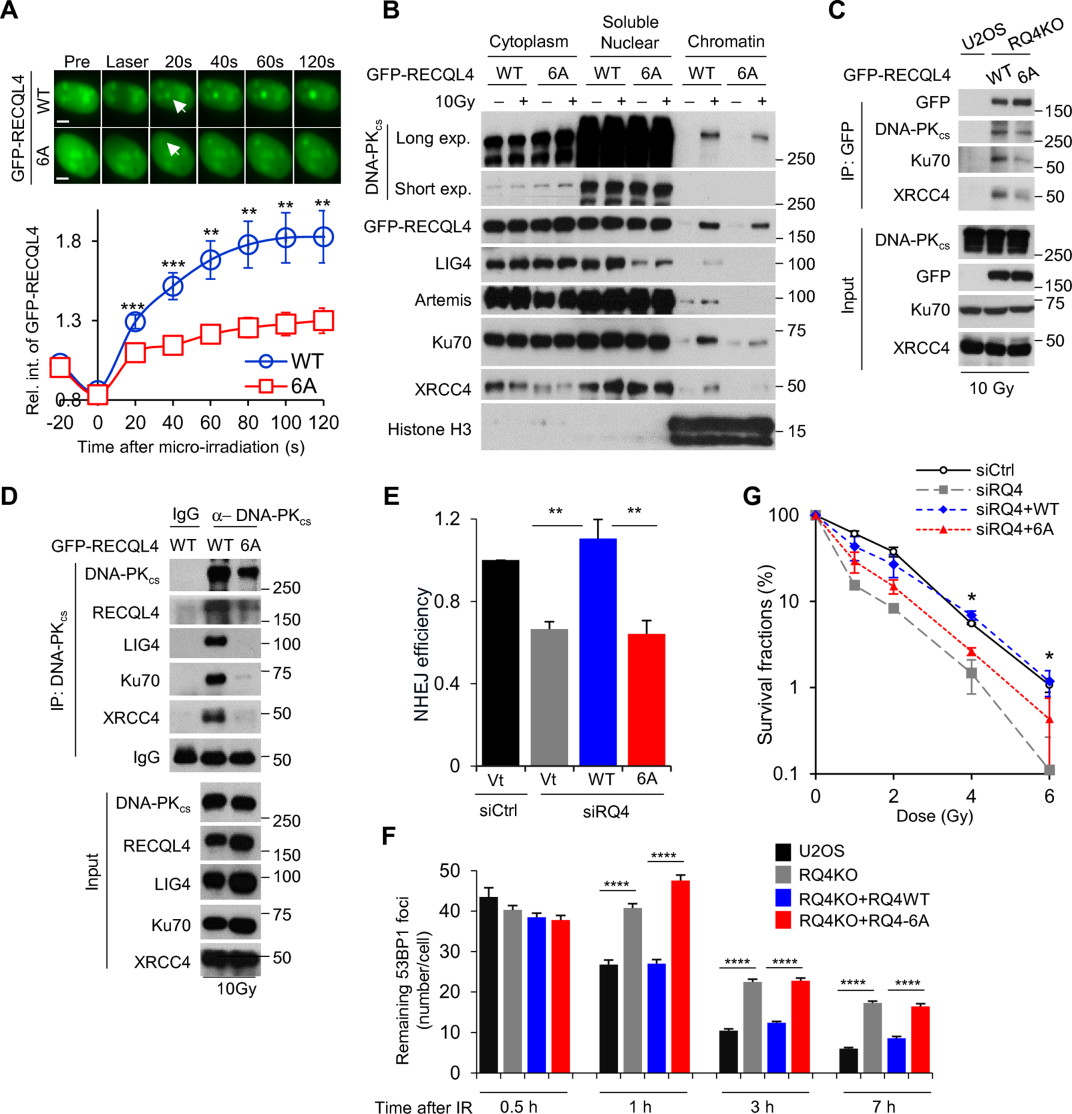
DNA-PK_cs_-mediated phosphorylation of RECQL4 promotes NHEJ. (**A**) Ablating the DNA-PK_cs_-mediated phosphorylation sites on RECQL4 significantly reduces the recruitment of RECQL4 to laser-generated DSBs. Relative fluorescent intensity of GFP-tagged wild-type RECQL4 (WT) and phosphorylation-null mutant RECQL4 (6A) following micro-irradiation are presented as mean ± SEM from 9 WT and 15 6A cells. (**B**) Blocking DNA-PK_cs_-dependent RECQL4 phosphorylation sites attenuates recruitment of NHEJ factors to the chromatin fraction following DNA damage. U2OS RECQL4 knockout cells stably expressing GFP-tagged WT or 6A were mock treated or irradiated with 10 Gy and allowed to recover for 10 min. Subsequently, cytoplasmic, soluble nuclear, and chromatin fractions were isolated for immunoblotting to examine the recruitment of proteins listed in the figure to the chromatin after irradiation. (**C**) RECQL4 phosphorylation modulates the IR-induced interactions between RECQL4 and NHEJ factors. U2OS RECQL4 knockout cells expressing GFP-tagged WT or 6A RECQL4 were irradiated with 10 Gy and allowed to recover for 10 min. GFP-tagged RECQL4 proteins were immunoprecipiated using a GFP antibody, the samples were resolved via SDS-PAGE, and interactions between RECQL4 and the core NHEJ factors DNA-PK_cs_, Ku70 and XRCC4 was determined via immunoblotting. (**D**) Ablating DNA-PK_cs_-mediated phosphorylation sites on RECQL4 decreases the interactions between the core NHEJ factors. U2OS RECQL4 knockout cells expressing GFP-tagged WT or 6A RECQL4 were irradiated with 10 Gy and allowed to recover for 10 min. DNA-PK_cs_ was immunoprecipiated, the samples were resolved via SDS-PAGE, and interactions between DNA-PK_cs_ and the core NHEJ factors Ku70, XRCC4 and XLF and RECQL4 was determined via immunoblotting. (**E**) RECQL4 phosphorylation promotes NHEJ-mediated DSB repair. Endogenous RECQL4 was depleted using *RECQL4* siRNAs in U2OS cells stabilizing the NHEJ GFP reporter assay EJ5 and subsequently the cells were transiently transfected with empty vector or siRNA-resistant plasmids that express 3XFLAG-tagged WT or 6A RECQL4. NHEJ-mediated DSB repair was evaluated using the GFP-based reporter assay. Student's t-test (two-sided) was performed to assess statistical significance (** *P*< 0.01). (**F**) IR-induced 53BP1 foci resolution is attenuated in 6A cells compared to WT in G1 cells. U2OS parental (U2OS) and RECQL4 knockout (RQ4KO) cells as well as RECQL4 knockout cells stably expressing wild type RECQL4 (RQ4KO + RQ4WT) or phosphorylation mutant (RQ4KO + RQ4-6A) were irradiated with 2 Gy of γ-rays and 53BP1 foci formation and resolution was assessed 0.5, 1, 3 and 7 h post-IR. Remaining 53BP1 foci at each time point were calculated in over 50 Cyclin A-negative cells and the data are presented as Mean ± SEM. Student's t-test (two-sided) was performed to assess statistical significance (**** *P*< 0.0001). (**G**) Colony formation assays were performed to compare the radiation sensitivities of U2OS cells, U2OS cells in which endogenous RECQL4 was depleted using *RECQL4* siRNAs, and U2OS RECQL4 knockdown cells complemented with 3XFLAG-tagged WT or 6A RECQL4. Cells were left cycling, irradiated at the indicated doses, and plated for analysis of survival and colony-forming ability. Student's t-test (two-sided) was performed to assess statistical significance (* *P*< 0.05).

We next aimed to elucidate the functionality of the RECQL4 phosphorylation sites. First, we assessed if blocking RECQL4 phosphorylation affects the dynamics of RECQL4 at laser-generated DSBs. As shown in Figure 6A, recruitment of GFP-tagged RECQL4 is attenuated when the phosphorylation sites are ablated compared to the wild-type protein. Furthermore, accumulation of RQ4-6A to the chromatin fraction following DNA damage was decreased compared to wild-type RECQL4 (Figure 6B). As RECQL4 promotes stabilization of the NHEJ machinery at DSBs, we examined if recruitment of NHEJ core factors to the chromatin fraction following DNA damage was diminished in RQ4-6A cells. We observed a marked decrease in DNA-PK_cs_, Ku70, LIG4, XRCC4 and Artemis recruitment to damaged chromatin in RQ4-6A cells compared to wild-type cells (Figure 6B). To support this, we used co-immunoprecipitation assays to assess if ablating RECQL4 phosphorylation affects the protein-protein interactions between the NHEJ factors. GFP-tagged RQ4-6A co-immunoprecipitated less of the NHEJ core factors, including DNA-PK_cs_, Ku70 and XRCC4, compared to wild-type RECQL4 (Figure 6C). Moreover, a marked decrease in Ku70, LIG4, XRCC4, and RECQL4 co-immunoprecipating with DNA-PK_cs_ was observed in RQ4-6A cells compared to RECQL4 wild-type cells (Figure 6D). These data show that DNA-PK-dependent phosphorylation of RECQL4 at the six N-terminal sites is important for the stabilization of the NHEJ machinery at DSBs. To investigate the consequence of blocking RECQL4 phosphorylation, we examined NHEJ efficiency using a GFP reporter assay. RECQL4 knockdown results in a significant decrease in NHEJ, which is corrected when complemented with wild-type RECQL4 (Figure 6E and Supplementary Figure S7A). Cells expressing the RQ4-6A protein have a similar decrease in NHEJ efficiency as the RECQL4 knockdown cells (Figure 6E). In addition, we measured the kinetics of 53BP1 foci resolution in G1 cells following IR as an indirect assay for NHEJ (65). As shown in Figure 6F, there is no difference in 53BP1 foci induction/resolution at each of the time points post-IR in the U2OS parental cells and the RQ4KO cells complemented with wild-type RQ4. However, a significant decrease in 53BP1 foci resolution is observed in RQ4KO cells and RQ4KO cells complemented with RQ4-6A at 1, 3 and 7h post-IR. These data illustrate that the DNA-PK phosphorylation sites are important for RECQL4′s role in promoting NHEJ-mediated DSB repair. The specificity of RECQL4 phosphorylation at these sites for NHEJ is supported by data showing that cells expressing RQ4-6A are not required for HR (Supplementary Figure S8). Finally, cells expressing RQ4-6A are radiosensitive compared to wild-type RECQL4, illustrating that phosphorylation of these sites are required for the cellular response to DNA damage (Figure 6G and Supplementary Figure S7B). Collectively, these data show that DNA-PK_cs_-dependent phosphorylation of RECQL4 promotes NHEJ by supporting the stabilization of the NHEJ machinery at DSBs.

## DISCUSSION

The DNA-PK complex is a first responder to DSBs, as both Ku70/80 and DNA-PK_cs_ localize to DSBs within seconds upon their generation (5). RECQL4 is also recruited to laser-induced DSB within 10 seconds, making it a candidate as an early responder to DNA damage (47,49). In this study, we aimed to elucidate RECQL4′s functions as an early responder to DSBs. The NHEJ factor DNA-PK_cs_, was found in a screen designed to identify RECQL4-interacting proteins at early time points following irradiation, suggesting that RECQL4 modulates DNA-PK_cs_. RECQL4 is not required for the recruitment of Ku70/80 or DNA-PK_cs_ to laser-generated DSBs, indicating that it does not regulate sensing of the DSB ends by the DNA-PK complex, but likely functions as an effector protein in the DDR. This is supported by our data showing that RECQL4 is required for the stabilization of the NHEJ machinery at DSBs and promotes DNA-PK-dependent DNA end bridging. Once RECQL4 is recruited to DSBs, there is reciprocal regulation between RECQL4 and DNA-PK_cs_ as DNA-PK_cs_ phosphorylates RECQL4 on six residues in the N-terminus of the proteins. This phosphorylation promotes the accumulation of RECQL4 to DSBs and stabilizes the NHEJ complex to support NHEJ.

This study adds to the growing list of reports showing that RECQL4 functions in pathways required for the repair of DSBs. RECQL4 coordinates NHEJ and HR in a cell cycle-dependent manner. Specifically, it preferentially interacts with the NHEJ factor DNA-PK and the HR factors MRE11-RAD50-NBS1 (MRN) and CtIP in the G1 and S/G2 phase of the cell cycle, respectively (53). MRE11 mediates the recruitment of RECQL4 to DSBs and this is regulated by phosphorylation of RECQL4 by CDK1 and CDK2 (49,53). RECQL4 then modulates DNA end resection by promoting the recruitment of the MRN accessory factor CtIP to DSBs and via its own helicase activity (49). In this study, we also examined if the DNA-PK and MRE-dependent recruitment of RECQL4 to DSBs is mutually exclusive. We found accumulation of RECQL4 to DSBs is more attenuated in cells treated with the DNA-PK_cs_ inhibitor NU7441 than MRE11 siRNAs, suggesting that DNA-PK plays a greater role in mediating the recruitment of RECQL4 to DSBs. To our surprise, attenuation of RECQL4 recruitment by MRE11 knockdown and DNA-PK inhibition was not additive as accumulation of RECQL4 to DSBs is similar in the NU7441 treated cells and the cells co-treated with MRE11 siRNA + NU7441. We speculate this could be due to two possibilities. First, this experiment was performed using an asynchronous population of cells and it is possible that a greater portion of the micro-irradiated co-treated (NU7441 + MRE11 siRNA) cells were in G1 phase of the cell cycle, which skewed the data collection in favor of the DNA-PK-dependent regulation of RECQL4 recruitment to DSBs. Second, DNA-PK_cs_ kinase activity promotes chromatin decondensation following IR-induced DNA damage to facilitate the rapid recruitment of the DNA damage response proteins, including the MRN complex, to DSBs, which may influence the MRE11-dependent recruitment of RECQL4 to DSBs (15). Finally, it has also been reported that RECQL4 regulates the choice between MMEJ and SSA. Cells expressing RECQL4 in which the C-terminal domain was deleted exhibit increased error-prone SSA activity and decreased MMEJ activity and ectopic expression of RECQL4 increased HR and MMEJ but repressed SSA (54). RECQL4 promoting HR and MMEJ is likely due to its ability to stimulate the initiation of DNA end resection. Collectively, these studies illustrate that RECQL4 functions in multiple pathways required for the repair of DSBs.

As RECQL4 positively influences multiple DSB repair pathways, a key question is how it coordinates these functions. We postulate that the critical event is phosphorylation of RECQL4. Previously, it was reported that CDK1 and CDK2 phosphorylate RECQL4 on serines 89 and 251 during S/G2 phases of the cell cycle (53). Phosphorylation of RECQL4 on these residues promote the interaction between RECQL4 and MRE11 and stimulates MRE11-dependent DNA end resection and HR. In this report, we show that DNA-PK_cs_ phosphorylates RECQL4 on six residues (S27, S101, T116, S180, S326 and T336) *in vitro*. Mutating these sites to alanine to ablate phosphorylation results in a marked attenuation of RECQL4 recruitment to DSBs and decreases the stabilization of the NHEJ machinery at DSBs and the completion of NHEJ. We hypothesize that RECQL4 phosphorylation is a switch that modulates DSB repair pathway choice. Ku binds to two-ended DSBs in all cell cycle phases (76,77). In G1 phase, RECQL4 is phosphorylated by DNA-PK_cs_ to induce NHEJ. In S/G2, the DNA-PK complex is removed from DNA ends through multiple mechanisms, resulting in decreased interaction between RECQL4 and DNA-PK. However, CDK1 and 2 are highly active in S/G2 phase and phosphorylate RECQL4 on S89 and S251 to stimulate DNA end resection and HR. We postulate that RECQL4 phosphorylation in the N-terminus of the protein allows it to tether to specific factors, including those required for NHEJ, HR, base excision repair, and pre-replicative complex required for DNA replication (78,79).

**Figure 7.**
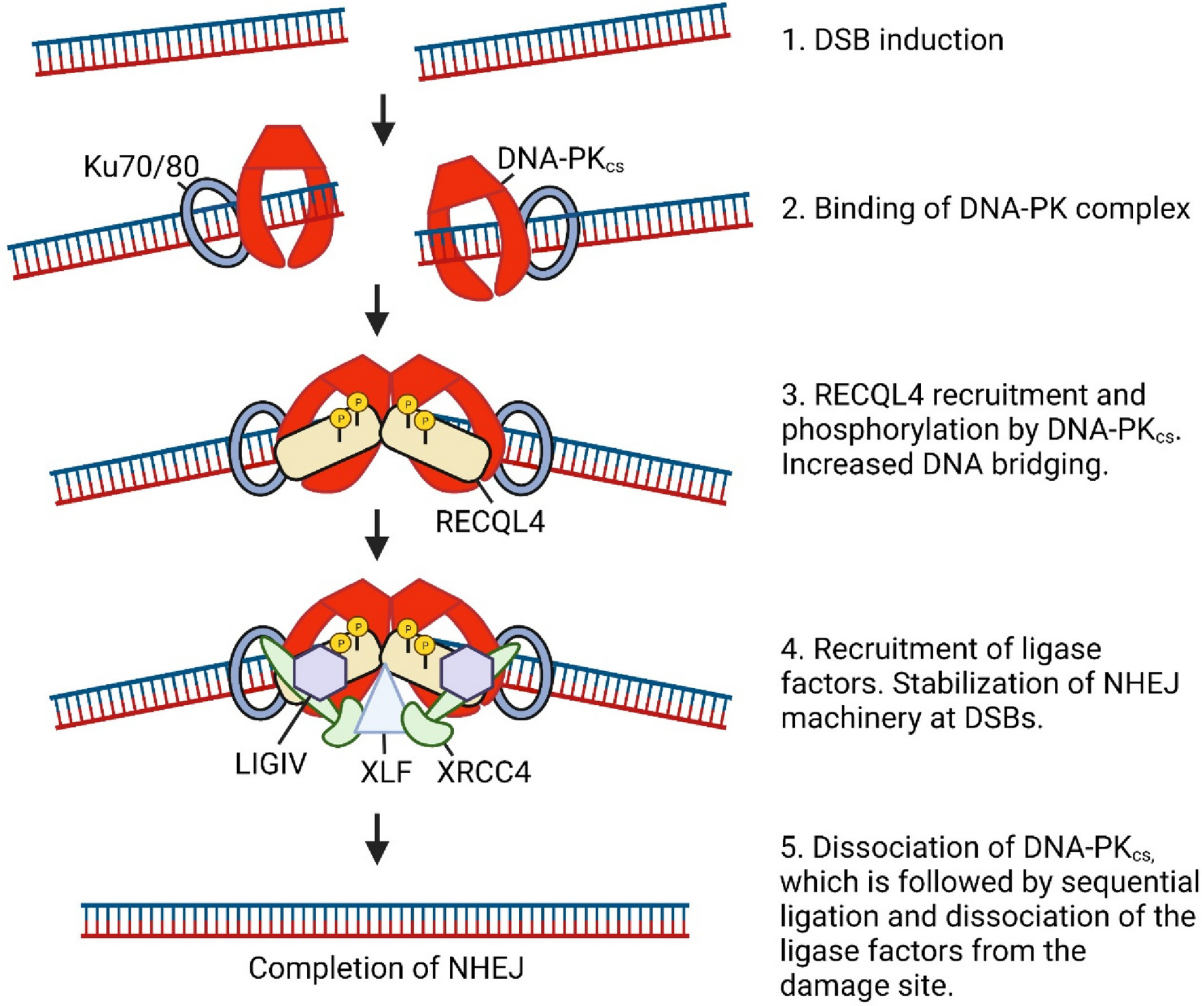
Model for the involvement of RECQL4 in NHEJ. Following induction of a DSB, the DNA-PK complex binds to the DSB ends. RECQL4 is then recruited to the DSB and phosphorylated by DNA-PK_cs_ on six residues in its N-terminus, which increases DNA-PK-mediated DNA end bridging. The ligase factors are recruited and RECQL4 in conjunction with DNA-PK stabilizes the NHEJ long range synaptic complex. Subsequently, DNA-PK_cs_ dissociates, the DSB is ligated following formation of the short range synaptic complex, and the rest of the NHEJ machinery detaches following completion of NHEJ. The model was generated with BioRender.com.

Based on the data presented in this manuscript and in the literature, we present the following model for the function of RECQL4 in NHEJ (Figure 7). Following induction of a two-ended DSB, the Ku heterodimer quickly binds to the DNA ends, which it protects from non-specific process, and subsequently it recruits DNA-PK_cs,_ and its kinase activity is stimulated. RECQL4 is recruited to DSBs, and it promotes DNA end bridging by the DNA-PK complex. It is likely this is due to a scaffolding effect, as the helicase activity is not required for RECQL4′s ability to promote stabilization of the NHEJ machinery at DSBs. Subsequently, RECQL4 is phosphorylated by DNA-PK_cs_ and we postulate this induces a confirmation change in RECQL4 that assists it in promoting the stabilization of the DNA-PK complex at DSBs in order to allow efficient recruitment of the core NHEJ factors to DSBs. It should be noted that loss of RECQL4 and DNA-PK_cs_ phosphorylation results in a significant, but modest decrease in NHEJ efficiency and radioresistance. We postulate that RECQL4 supports NHEJ by promoting the NHEJ long-range synaptic complex via stabilization of the NHEJ machinery at DSBs (67,69,80). RECQL4 has strong single strand annealing activity and this activity is not lost in the RECQL4 helicase-dead mutant (RQ4KM) (36); therefore, it is also possible that RECQL4 promotes NHEJ for a subset of DSBs, such as those with frayed DSB ends, in order to allow recruitment of the NHEJ machinery at DSBs. DNA-PK_cs_ then dissociates to allow the formation of the NHEJ short-range synaptic complex, DNA end processing occurs if it is required, and sequential ligation of the broken DNA strands. Finally, there is dissociation of the NHEJ machinery from the DNA damage site and NHEJ is completed.

## DATA AVAILABILITY

All study materials will be made available to other researchers. Please contact Anthony J. Davis (anthony.davis@utsouthwestern.edu) for reagents.

## Supplementary Material

gkac375_Supplemental_Files
