# Supplementary material for: DNA-PKcs-dependent phosphorylation of RECQL4 promotes NHEJ by stabilizing the NHEJ machinery at DNA double-strand breaks
Source: Nucleic Acids Res. 2022 May 17;50(10):5635–51. doi: 10.1093/nar/gkac375 (PMC9178012; doi:10.1093/nar/gkac375)
Supplement: gkac375_Supplemental_Files [file gkac375_supplemental_files.zip › Supplemental Figures R2.pdf]

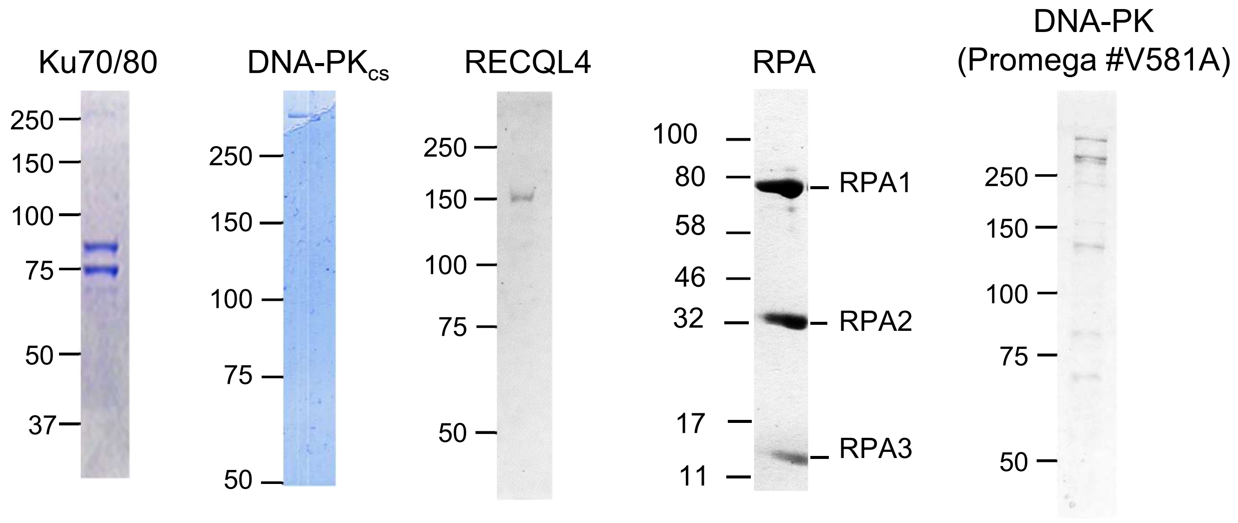

Sup. Fig. 1 Coomassie blue staining of the proteins used in this study.

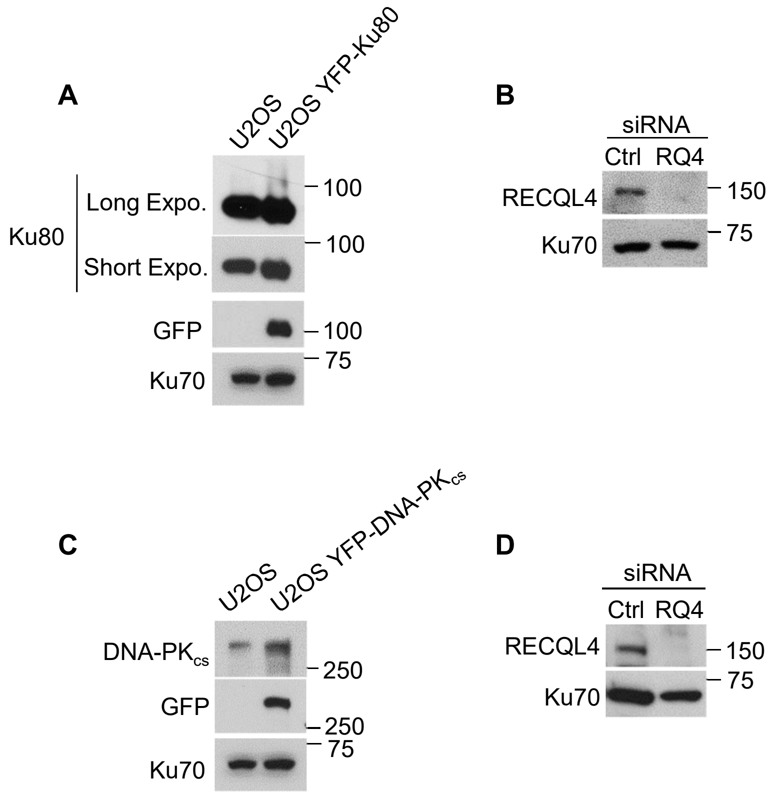

Sup. Fig. 2 Immunoblotting showing knockdown of RECQL4 in U2OS cells stably expressing YFP-Ku80 and YFP-DNA-PK$_{cs}$. **(A)** Expression of endogenous Ku80 and YFP-tagged Ku80 in U2OS cells and U2OS cells stably expressing YFP-Ku80. **(B)** Knockdown of RECQL4 by *RECQL4* siRNA in U2OS cells stably expressing of YFP-Ku80. **(C)** Expression of endogenous DNA-PK$_{cs}$ and YFP-tagged DNA-PK$_{cs}$ in U2OS cells and U2OS cells stably expressing YFP-DNA-PK$_{cs}$. **(D)** Knockdown of RECQL4 by *RECQL4* siRNA in U2OS cells stably expressing YFP-DNA-PK$_{cs}$.

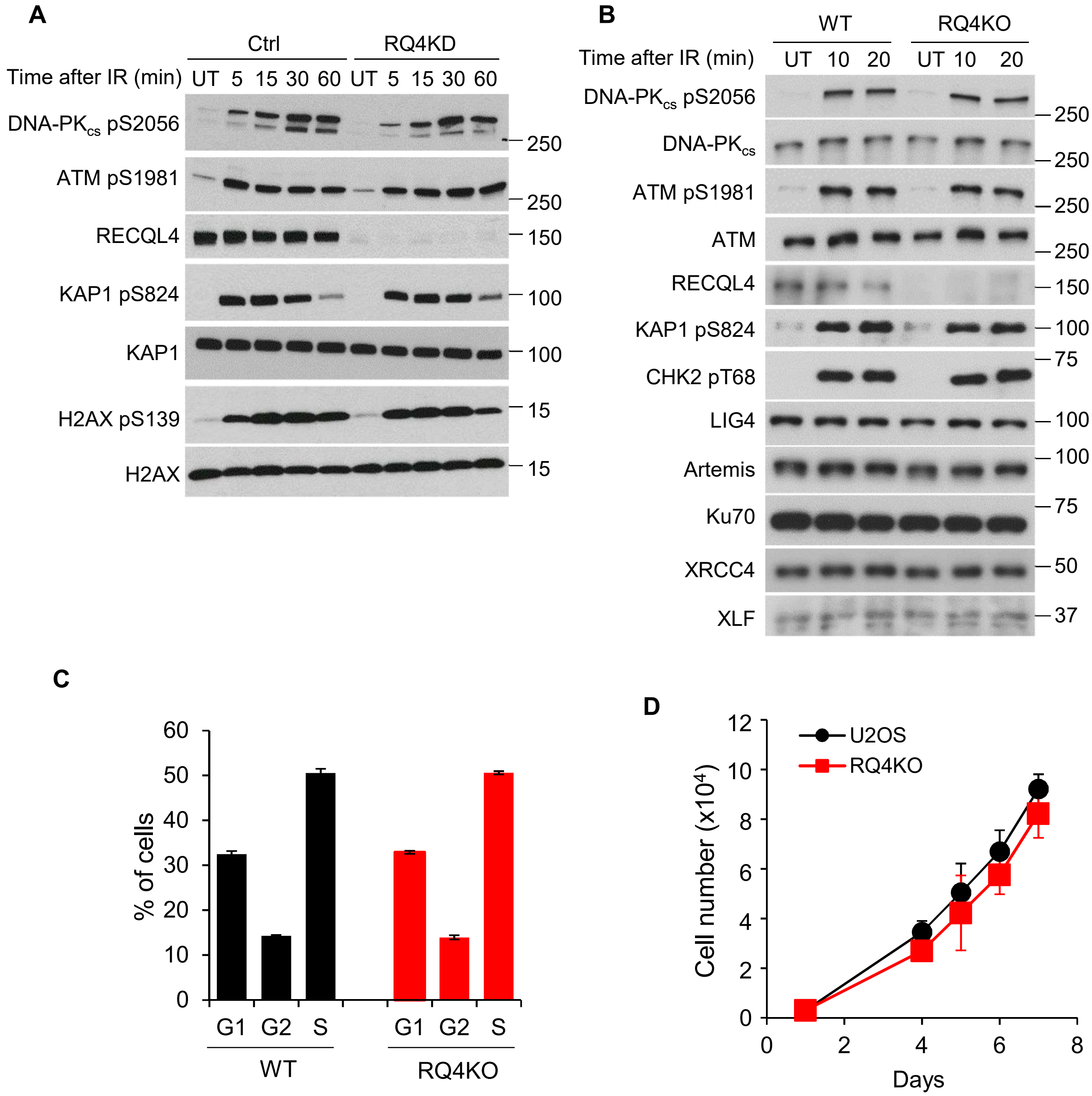

Sup. Fig. 3 Loss of RECQL4 does not affect the initiation of DNA damage response signaling after irradiation and does not alter cell cycle distribution and cell proliferation rate. U2OS cells treated with control siRNAs (Ctrl) or *RECQL4*-directed siRNAs (RQ4KD) **(A)** and U2OS wild-type and RECQL4 knockout (RQ4KO) **(B)** were mock irradiated or irradiated with 10 Gy and allowed to recover for the indicated time. Whole cell lysates were obtained, and immunoblotting was performed to assess the phosphorylation status of DNA-PK$_{cs}$ at serine 2056, ATM at serine 1981, KAP1 at serine 824, and H2AX at serine 139. **(C)** Cell cycle distribution of U2OS wild type cells (WT) and RECQL4 knockout cells (RQ4KO) were measured by flow cytometry after propidium iodide staining. **(D)** Proliferation rate of U2OS wild type cells (WT) and RECQL4 knockout cells (RQ4KO).

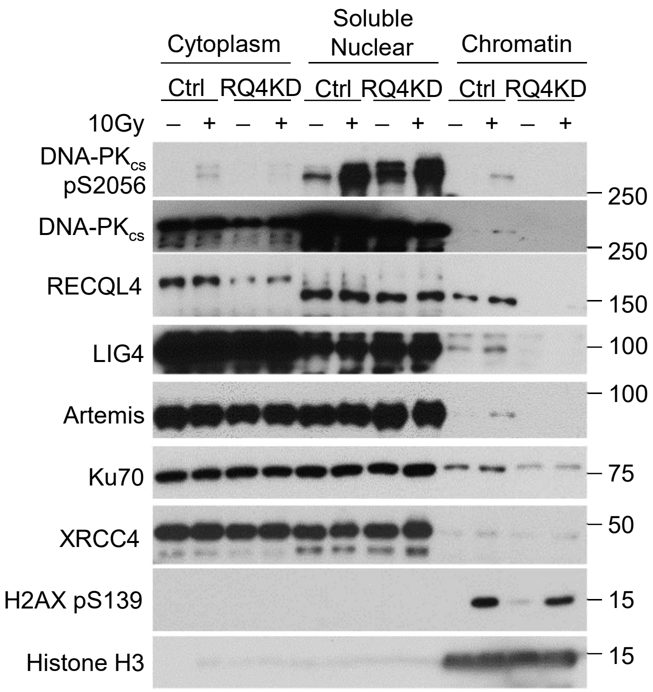

Sup. Fig. 4 Recruitment of NHEJ core factors to chromatin after IR is attenuated in RECQL4 knockdown cells. U2OS cells treated with control siRNAs (Ctrl) or *RECQL4*-directed siRNAs (RQ4KD) were mock-treated or irradiated with a dose of 10 Gy and allowed to recover for 10 minutes. Subsequently, cytoplasmic, soluble nuclear, and chromatin fractions were isolated for immunoblotting to examine the recruitment of proteins listed in the figure to the chromatin after irradiation.

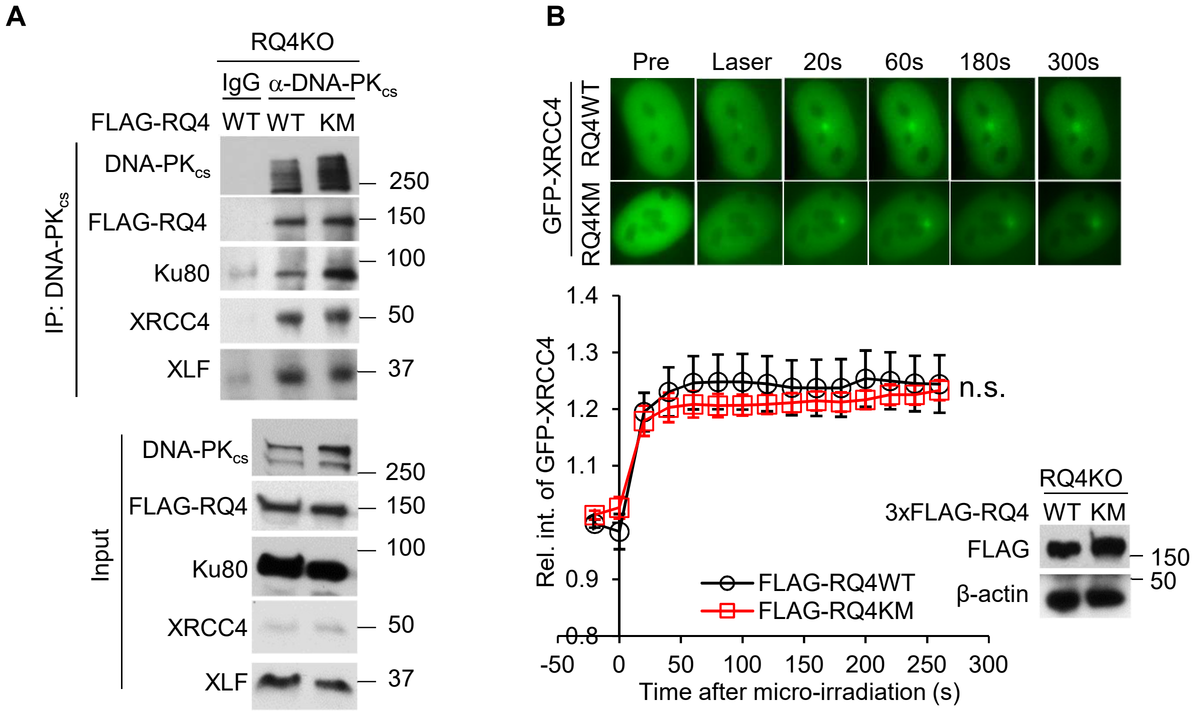

Sup. Fig. 5 Helicase activity is not required for the ability of RECQL4 to promote the stabilization of the NHEJ machinery to DSBs. **(A)** Interaction between DNA-PK$_{cs}$ and canonical NHEJ factors following IR is similar in RECQL4 knockout U2OS cells stably expressing 3XFLAG-tagged RECQL4 wild-type (WT) and a RECQL4 helicase-dead mutant (KM). Specifically, the cells were irradiated with a dose of 10 Gy and allowed to recover for 10 minutes. Then, DNA-PK$_{cs}$ was immunoprecipitated, and interactions between DNA-PK$_{cs}$ and the NHEJ factors (Ku80, XRCC4, and XLF) was assessed via immunoblotting. **(B)** Inactivation of the helicase activity of RECQL4 does not disrupt the recruitment of GFP-XRCC4 to laser-induced DSBs. GFP-XRCC4 was transiently expressed in RECQL4 knockout U2OS cells stably expressing 3XFLAG-tagged RECQL4 wild type (FLAG-RQ4WT) or helicase-dead mutant (FLAG-RQ4KM), and recruitment of GFP-XRCC4 to laser-induced DSBs was monitored. 13 WT cells and 11 KM cells were measured, and the results are presented as mean±SEM. Statistical analysis via Student's t test found that XRCC4 recruitment to DSBs all time points was not statistically (n.s.) different between the FLAG-RQ4WT and FLAG-RQ4KM cell lines. Bar indicates 5 μm.

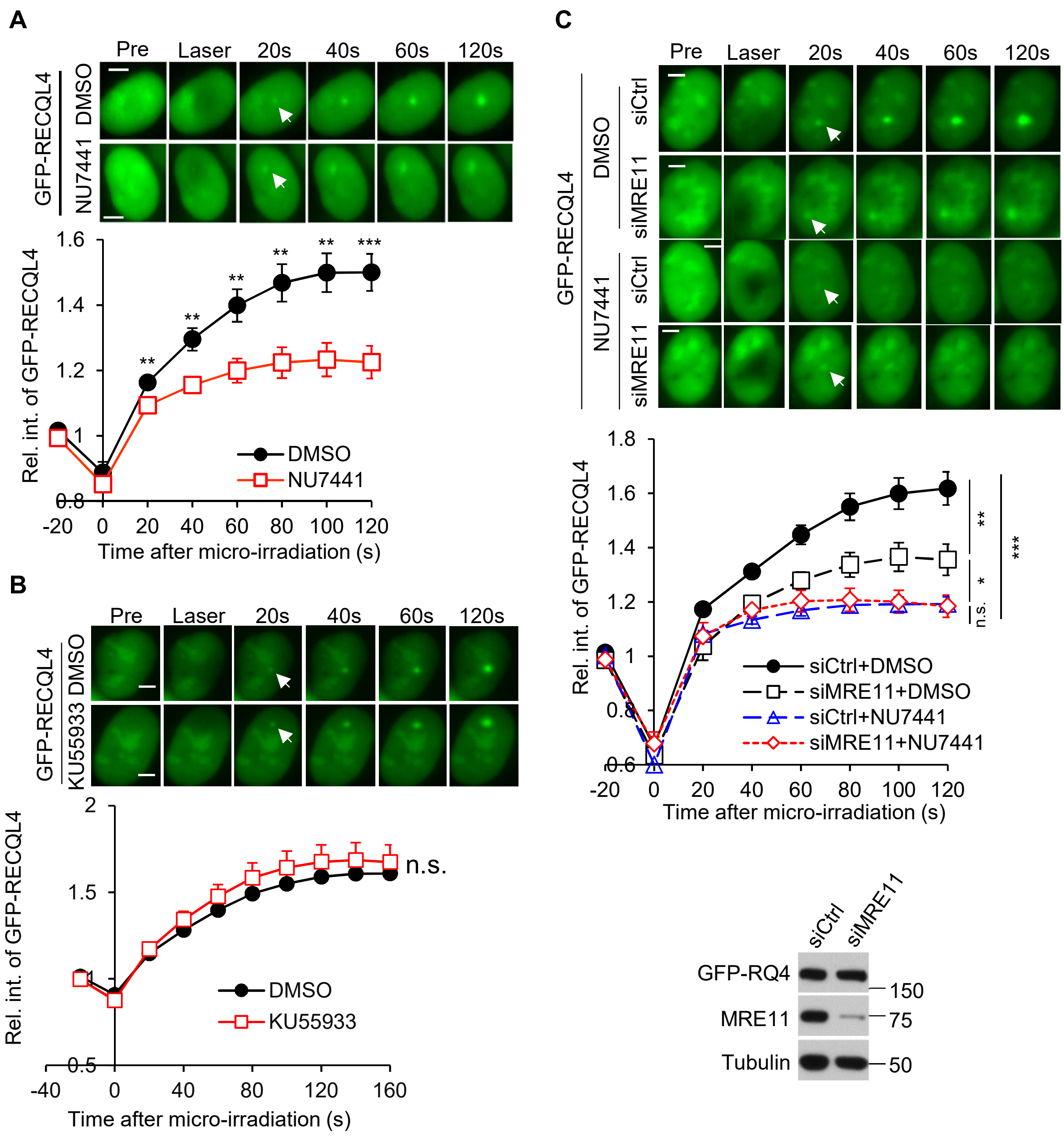

Sup. Fig. 6 Recruitment of RECQL4 to laser-induced DSBs is regulated by DNA-PK$_{cs}$ and MRE11, but not ATM. U2OS RECQL4 knockout cells stably expressing GFP-tagged RECQL4 were pretreated with DMSO **(A and B)**, 3 μM NU7441 **(A)**, or 10 μM KU55933 **(B)** for 2 hours, and subsequently laser micro-irradiation assays were performed. Relative fluorescent intensity of GFP-tagged REQL4 following micro-irradiation are presented as mean ± standard error of the mean (SEM) in **(A)** and **(B)**. Samples analyzed were 8 for DMSO-treated and 12 NU7441-treated cells in **(A)** and 9 DMSO-treated cells and 10 KU55933-treated cells in **(B)**. **(C)** RECQL4 knockout U2OS cells stably expressing GFP-tagged RECQL4 were transfected with control siRNA (siCtrl) or *MRE11* siRNA (siMRE11) and two days later were treated with 3 μM NU7441 or DMSO for 2 hours, and subsequently laser micro-irradiation assays were performed. Knockdown of MRE11 was assessed by immunoblotting (see bottom panel). Relative fluorescent intensity of GFP-tagged RECQL4 following micro-irradiation are presented as mean ± SEM from 10 siCtrl+DMSO cells, 9 siMRE11+DMSO cells, 14 siCtrl+NU7441 cells and 12 siMRE11+NU7441 cells. For all experiments, student's t-test (two-sided) was performed to assess statistical significance (* p<0.05, ** p<0.01 and *** p<0.001) and not significant (n.s.). Bar indicates 5 μm.

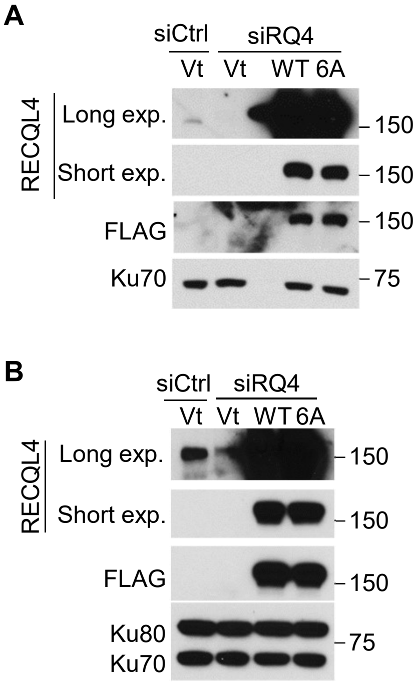

Sup. Fig. 7 Immunoblotting showing the knockdown of RECQL4 in EJ5 U2OS cells **(A)** and U2OS cells **(B)** and complementation with either wild-type (WT) or phosphorylation-dead (6A) RECQL4.

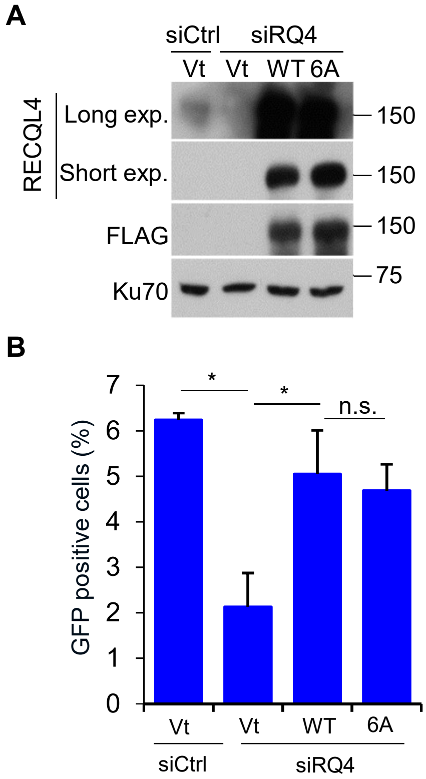

Sup. Fig. 8 DNA-PK_{cs}-mediated phosphorylation of RECQL4 does not affect RECQL4's role in promoting HR-mediated DSB repair. **(A)** Immunoblotting showing protein level of endogenous RECQL4 or 3XFLAG-tagged RQ4-WT and RQ4-6A proteins in DR-GFP U2OS cells treated with control siRNA or *RECQL4* siRNA. **(B)** Percentage of GFP-positive cells which represent the efficiency of HR-mediated DSB repair in the four cell lines. The results are presented as mean ± SEM with p-value from three repeats. *,<0.05; n.s., not significant.
